# Phytoplankton settling quality has a subtle but significant effect on sediment microeukaryotic and bacterial communities

**DOI:** 10.1038/s41598-021-03303-x

**Published:** 2021-12-15

**Authors:** Séréna Albert, Per Hedberg, Nisha H. Motwani, Sara Sjöling, Monika Winder, Francisco J. A. Nascimento

**Affiliations:** 1 Department of Ecology, Environment and Plant Sciences, Stockholm University, Svante Arrhenius 20A, 106 91 Stockholm, Sweden; 2 Department of Environmental Science, School of Natural Sciences, Technology and Environmental Studies, Södertörn University, Huddinge, Sweden; 3 Baltic Sea Centre, Stockholm University, Stockholm, Sweden

**Keywords:** Ecology, Environmental sciences, Ocean sciences

## Abstract

In coastal aphotic sediments, organic matter (OM) input from phytoplankton is the primary food resource for benthic organisms. Current observations from temperate ecosystems like the Baltic Sea report a decline in spring bloom diatoms, while summer cyanobacteria blooms are becoming more frequent and intense. These climate-driven changes in phytoplankton communities may in turn have important consequences for benthic biodiversity and ecosystem functions, but such questions are not yet sufficiently explored experimentally. Here, in a 4-week experiment, we investigated the response of microeukaryotic and bacterial communities to different types of OM inputs comprising five ratios of two common phytoplankton species in the Baltic Sea, the diatom *Skeletonema marinoi* and filamentous cyanobacterium *Nodularia spumigena*. Metabarcoding analyses on 16S and 18S ribosomal RNA (rRNA) at the experiment termination revealed subtle but significant changes in diversity and community composition of microeukaryotes in response to settling OM quality. Sediment bacteria were less affected, although we observed a clear effect on denitrification gene expression (*nirS* and *nosZ*), which was positively correlated with increasing proportions of cyanobacteria. Altogether, these results suggest that future changes in OM input to the seafloor may have important effects on both the composition and function of microbenthic communities.

## Introduction

Organic matter (OM) export from the pelagic zone is normally the main source of energy for benthic organisms living in coastal aphotic sediments^1,2^. OM export to the seafloor varies in terms of quantity and quality, and both factors have been identified as key drivers of benthic diversity, activity and community composition^2,3^. Pelagic OM sedimentation follows a marked seasonal pattern. In many temperate coastal aphotic sediments, diatoms contribute substantially to the downward export of OM in the spring. They sink rapidly throughout the water column and reach the sediment in a relatively fresh state^4^. As such, spring bloom-derived OM constitutes the main input of the year for benthic organisms^5^. During the summer, phytoplankton communities are often dominated by nitrogen-fixing cyanobacteria, which can form extensive blooms at the water surface^6^. Cyanobacteria have lower sedimentation rates than diatoms, and as a consequence, undergo more mineralization in the water column, reaching the seafloor in a more degraded state^4^. Anthropogenic pressures, including global climate change, have triggered rapid changes in environmental conditions in many coastal ecosystems, notably the Baltic Sea^7^. As a result, long-term trends show decreasing diatom biomass in the spring, coupled with a larger occurrence of dinoflagellates^8^. In parallel, warmer summer temperatures and excess phosphorus (P) availability are suggested to increase the abundance of nitrogen-fixing cyanobacteria^9^. Given the intricate links between pelagic and benthic systems, changes in the composition and sedimentation of phytoplankton OM are likely to have substantial effects on soft-sediment communities in terms of biodiversity and capacity to sustain crucial ecosystem processes^1,10^.

Microeukaryotes, broadly including meiofaunal metazoans (organisms between 40 µm and 1 mm, e.g. nematodes, platyhelminthes, copepods) and protists (e.g. ciliates, flagellates, Foraminifera), are particularly diverse and hold a central position in soft-sediments habitats^11^. They are important food sources for larger organisms, and interact closely with sediment bacteria^12^. Through particle reworking, grazing or excretion, microeukaryotes can influence bacterial diversity and activity, and therefore have the potential to alter benthic ecosystem processes, including OM mineralization and denitrification^12–14^. Bacteria can account for up to 90–99% of the total benthic biomass and control most of the carbon (C) and nitrogen (N) cycling in marine sediments^15^. Both microeukaryotes and bacteria utilize settled OM as food. Due to their quick turnover times, they can respond rapidly to OM pulses, such as the sedimentation of the spring bloom, by increasing their activity, biomass or abundance^2,16–18^.

The quantity of OM that settles on the seafloor influences metazoan meiofauna, ciliates and bacterial communities^17,19,20^. Pulses of fresh OM may for instance favor specific meiobenthic organisms that are well adapted to utilize this material^21^. In high quantities, OM loading can also have a negative impact on ciliate alpha diversity^20^. However, the importance of the quality of settling OM as a driver of benthic community composition and diversity has received less attention^3^. OM composition may change the trophic structure of nematode assemblages^22,23^, presumably reflecting adapted resource utilization based on morphological characteristics^24^. However, such responses are likely to be context-dependent, as nematodes can exhibit broad trophic plasticity, adapting their foraging to resource availability^25^. The capacity of a number of meiofauna species to use cyanobacteria as food has been previously demonstrated^26^, although growth was impaired compared to higher quality food such as diatoms^27^. Conversely, cyanobacteria can be a valuable resource for marine invertebrates, e.g. when used as a complement to other dietary sources^28^. It remains unclear how such effects, observed on a few species, might translate to the community scale. Likewise, few studies have addressed the effects of settling OM quality on bacterial communities in Baltic Sea soft-sediments^29^. In other marine systems, qualitative parameters such as fatty acid content or OM source have been found to drive significant changes in microbial community composition and diversity^19,30^. Structural changes could in turn alter the functional role played by benthic bacteria^31^. For instance, denitrification, the microbially-mediated reduction of nitrate to dinitrogen (N_2_) gas responded negatively to diatoms addition in sediment where nitrification and denitrification are tightly coupled^32^, while positive effects have been observed after cyanobacteria amendments^33^. Overall, it is still unclear how microeukaryotes and bacteria respond at the community level to gradual changes in the composition and quality of settling OM.

The response of microeukaryotic and bacterial communities to phytoplankton-derived OM in Baltic Sea soft sediments was explored in this study. Additionally, this study provided much needed insight into the effects of phytoplankton OM quality on denitrification, an important process that counteracts coastal eutrophication through the removal of bioavailable N. A mesocosm experiment was conducted, where OM inputs as a result of diatoms and cyanobacteria sedimentation were simulated in five different proportions to intact sediment cores. After 4 weeks, environmental RNA (eRNA) metabarcoding was used to investigate changes in biologically active communities of microeukaryotes (18S rRNA) and bacteria (16S rRNA). The expression of *nirS* and *nosZ* genes, respectively involved in the second and fourth steps of denitrification, was also quantified using real-time quantitative PCR (RT-qPCR). The following hypotheses were tested: (i) Differences in OM quality significantly change the diversity and community composition of metazoan meiofauna, protists and bacteria; and (ii) the expression of genes involved in denitrification, *nirS* and *nosZ,* is positively related to quantity of cyanobacterial OM that reaches the sediment. We expect our findings to provide a better insight into how future changes in OM settling composition might affect the structure and function of benthic communities.

## Results

### General description of the datasets

eRNA metabarcoding of 18S and 16S rRNA amplicons yielded a total of 4,806,244 and 2,507,807 reads, respectively (Supplementary Tables S1 and S2). A large number of reads from the 16S rRNA dataset were attributed to known bacterial phyla (89.1 ± 0.6%, Supplementary Fig. S1), while the known proportion was lower in the 18S rRNA dataset, with 73.3 ± 0.6% of reads assigned to an eukaryotic phylum (Supplementary Fig. S1). After filtering out non-target organisms, single- and doubletons, the microeukaryotes (18S) and bacteria (16S) rRNA datasets consisted of 3079 and 14,097 unique amplicon sequence variants (ASVs), respectively (Supplementary Tables S1 and S2).

Taxonomic information retrieved from rRNA metabarcoding reflects organisms that had a protein synthesis potential at the time of sampling^34^. eRNA approaches have therefore been used as a means of preferentially identifying the active fraction of communities^35^. We will therefore refer to “active” organisms in contrast to deceased ones, keeping in mind that dormant organisms may still contain large amounts of rRNA^34^.

**Figure 1:**
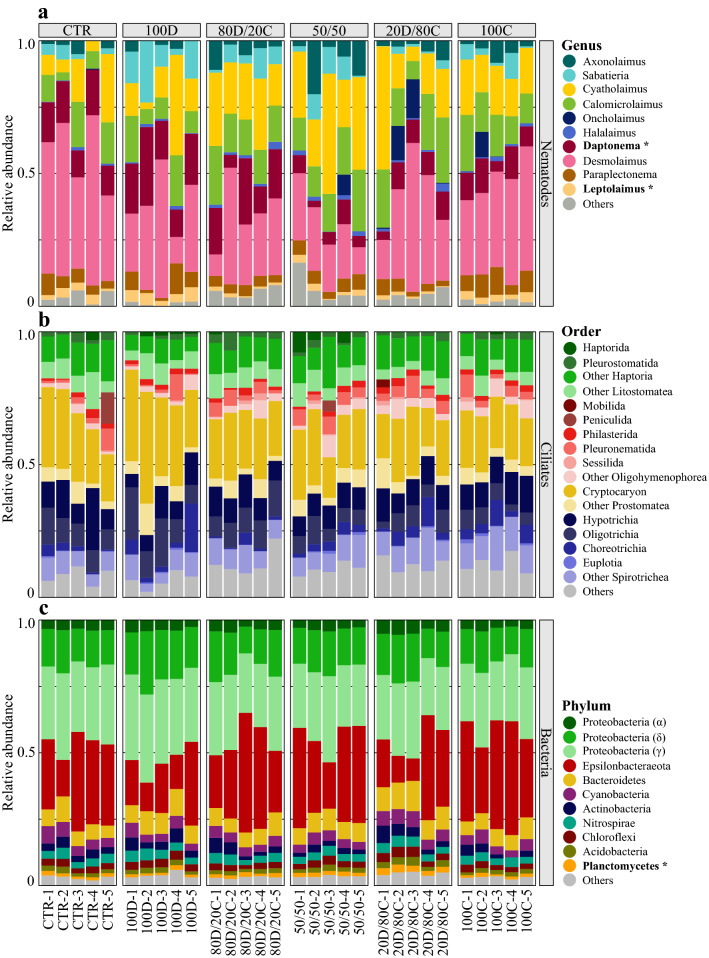
Taxonomic composition of (**a**) nematodes (18S rRNA, Genus level), (**b**) ciliates (18S rRNA, Order level) and (**c**) bacterial (16S rRNA, Phylum level) communities, displayed as ASVs relative abundances. Taxonomic groups showing significant differences in relative abundance across treatments after multiple testing correction are indicated in bold and with an asterisk (*). See Fig. 6 for labels on the x-axis.

Overall, ciliates (39.8%) and nematodes (27.6%) were the most abundant groups in our eRNA metabarcoding 18S rRNA dataset. Arthropods contributed 12.9% of total reads, but most ASVs were unclassified below phylum level. Remaining taxa comprised platyhelminthes, rotifers, and numerous other low-abundance groups. Nematode assemblages were dominated by *Desmolaimus* (32.1 ± 2.7%), *Cyatholaimus* (18.9 ± 2.0%) and *Calomicrolaimus* (14.3 ± 1.0%, Fig. 1a). Ciliate assemblages were dominated by the genus *Cryptocaryon* (23.7 ± 1.1%), other Haptoria (10.5 ± 0.5%) and other Hypotrichia (10.1 ± 0.6%, Fig. 1b). In our 16S rRNA dataset, the dominant bacteria phyla were Epsilonbacteraeota (dominated by the genus *Arcobacter*, 27.3 ± 2.0%), Proteobacteria (Gamma: 26.9 ± 0.6%, Delta: 15.1 ± 0.5%) and Bacteroidetes (7.3 ± 0.3%, Fig. 1c).

### Effects of OM quality on alpha diversity

**Figure 2:**
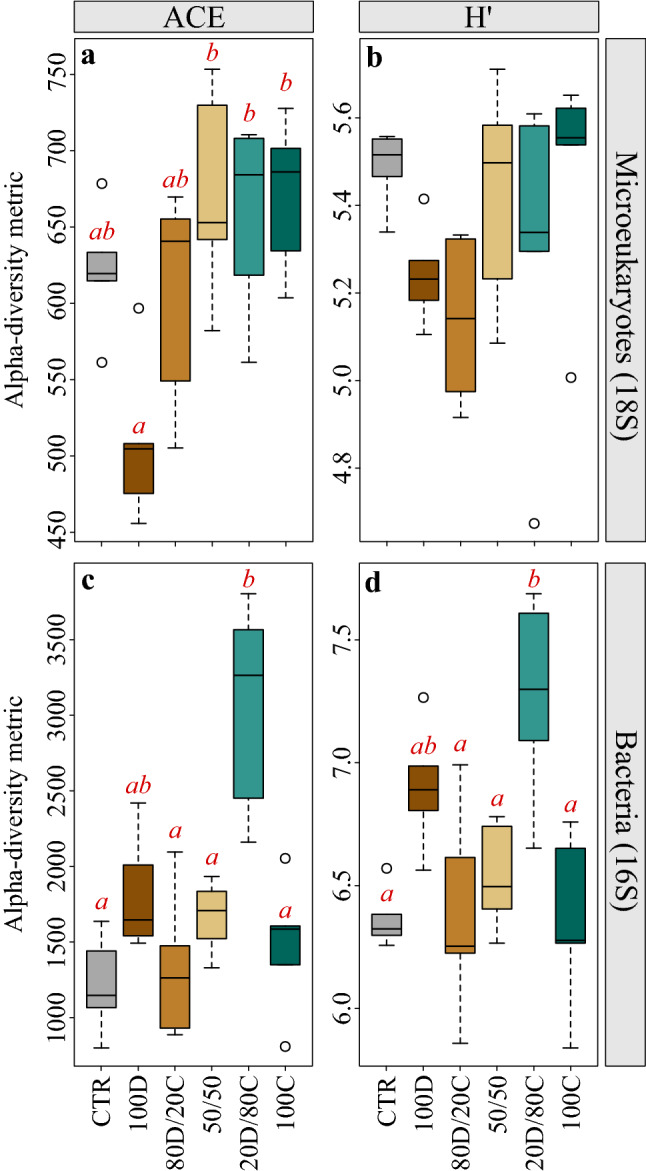
Alpha diversity indices (Abundance-based coverage estimator (ACE) and Shannon’s diversity index H′) for (**a**,**b**) microeukaryotes and (**c**,**d**) bacteria upon experiment termination. Red letter codes designate the significant differences across treatments based on one-way ANOVA and TukeyHSD post-hoc test results. See Fig. 6 for labels on the x-axis.

Rarefaction curves approached a plateau for all 18S and 16S rRNA samples, indicating that the abundant ASVs present in the ecosystem during the time of sampling were successfully retrieved (Supplementary Fig. S2). Among microeukaryotes, OM composition had a significant impact on alpha diversity (Fig. 2a,b), inducing lower species richness (Abundance-based coverage estimator, ACE) in the 100% diatom (100D) treatment compared to treatments with 50% cyanobacteria (50/50) and higher (ANOVA, F_5,24_ = 5.38, p = 0.002, Table 1). A similar trend was observed for the Shannon’s diversity index H′, although differences were not significant across treatments (ANOVA, F_5,24_ = 1.75, p = 0.162, Table 1). Further taxonomic partitioning of the microeukaryotes dataset revealed that the overall decrease in diversity in 100D was observed among both nematodes and ciliates (Supplementary Fig. S3). Nematodes richness was lower in 100D than in the 20% diatoms/80% cyanobacteria (20D/80C) treatment (ACE: ANOVA, F_5,24_ = 3.33, p = 0.02, Table 1), and ciliates Shannon’s diversity index H′ was lower in 100D compared to the 50/50 and 100% cyanobacteria (100C) treatments (ANOVA, F_5,24_ = 5.03, p = 0.003, Table 1). OM composition also had an effect on bacterial alpha diversity (Fig. 2c,d). Both ACE and Shannon’s diversity H′ indices were significantly higher in 20D/80C compared to all other treatments, except 100D (ACE: ANOVA, F_5,24_ = 7.51, p < 0.001; H′: ANOVA, F_5,24_ = 6.64, p < 0.001, Table 1).

**Table 1:** Summary of statistical tests.

	Target group		Variable	Test	Test statistic	r^2^	p	CTR	100D	80D/20C	50/50	20D/80C	100C
Alpha diversity	Eukaryotes (18S)	All	ACE	ANOVA	F_(5,24)_ = 5.38	**–**	**0.002**	*ab*	*a*	*ab*	*b*	*b*	*b*
			H′	ANOVA	F_(5,24)_ = 1.75	**–**	0.162	**–**					
		Nematodes	ACE	ANOVA	F_(5,24)_ = 3.33	**–**	**0.020**	*ab*	*a*	*ab*	*ab*	*b*	*ab*
			H′	ANOVA	F_(5,24)_ = 2.10	**–**	0.100	**–**					
		Ciliates	ACE	ANOVA	F_(5,24)_ = 2.40	**–**	0.067	**–**					
			H′	ANOVA	F_(5,24)_ = 5.03	**–**	**0.003**	*ab*	*a*	*ab*	*b*	*ab*	*b*
	Bacteria (16S)	All	ACE	ANOVA	F_(5,24)_ = 7.51	**–**	** < 0.001**	*a*	*ab*	*a*	*a*	*b*	*a*
			H′	ANOVA	F_(5,24)_ = 6.64	**–**	** < 0.001**	*a*	*ab*	*a*	*a*	*b*	*a*
Gene expression			*nirS*	Kruskal–wallis	H_(5)_ = 25.81	**–**	** < 0.001**	*ab*	*a*	*ab*	*abc*	*bc*	*c*
			*nosZ*	Kruskal–wallis	H_(5)_ = 26.18	**–**	** < 0.001**	*ab*	*ab*	*bc*	*bcd*	*cd*	*d*
Community composition	Eukaryotes (18S)	All	Sørensen distance matrix	Permanova (999)	Pseudo-F_(5,24)_ = 1.62	0.25	**0.003**	**–**					
		Nematodes	Sørensen distance matrix	Permanova (999)	Pseudo-F_(5,24)_ = 1.59	0.25	**0.028**	**–**					
		Ciliates	Sørensen distance matrix	Permanova (999)	Pseudo-F_(5,24)_ = 1.88	0.28	**0.001**	**–**					
	Bacteria (16S)	All	Sørensen distance matrix	Permanova (999)	Pseudo-F_(5,24)_ = 1.36	0.22	**0.003**	**–**					

Differences in alpha diversity indices (Abundance-based coverage estimator (ACE) and Shannon’s diversity index H′) were tested with one-way ANOVAs for the microeukaryotes (18S rRNA) and bacteria (16S rRNA) datasets as well as nematodes and ciliates separately. Difference in transcript copy numbers for the denitrification genes *nirS* and *nosZ* were tested with Kruskal–Wallis. Differences in community composition were tested with PERMANOVAs on the microeukaryotes (18S rRNA) and bacteria (16S rRNA) datasets. Significant p-values (< 0.05) are indicated in bold. The treatment groupings based on post-hoc tests are displayed as letter codes on the right of the table. See Fig. 6 for treatment labels.

### Effects of OM quality on community structure

**Figure 3:**
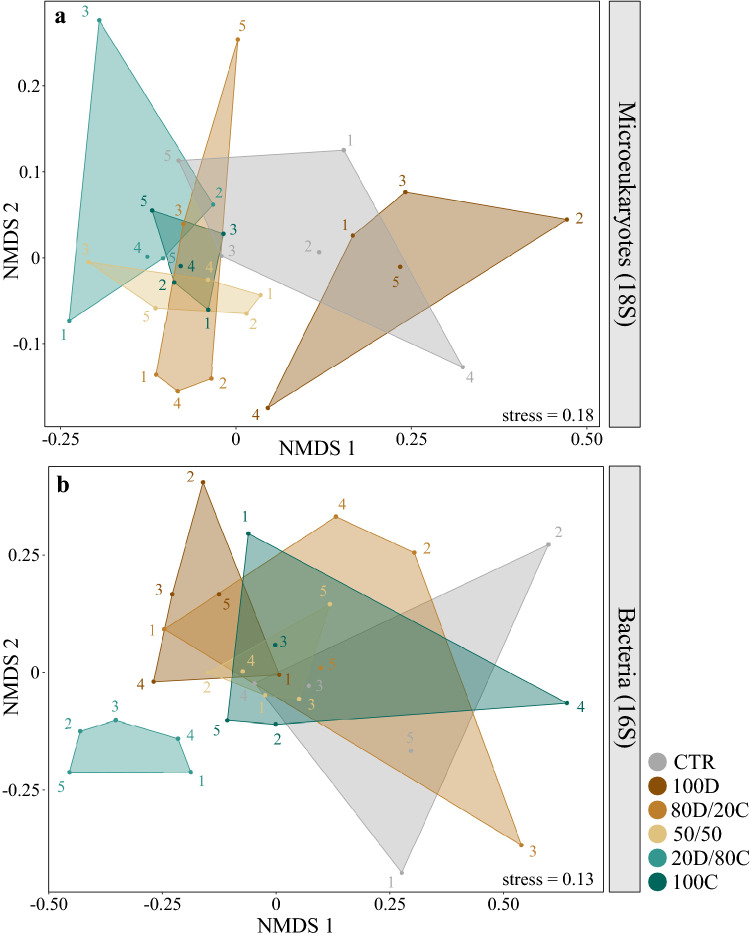
NMDS ordination plots based on Sørensen distance matrices of (**a**) microeukaryotic and (**b**) bacterial communities, calculated from 18 and 16S rRNA ASVs presence/absence, respectively. The stress value for each ordination is displayed in the bottom right corner. See Fig. 6 for treatment codes in the legend.

Microeukaryotic community structure was significantly affected by OM composition, with 25% of the variation explained by the different treatments (PERMANOVA, Pseudo-F_5,24_ = 1.62, r^2^ = 0.25, p = 0.003, Table 1). The NMDS ordination shows that the 100D treatment clustered separately from all cyanobacteria treatments, regardless of their proportion as OM input, while CTR overlapped with both groups (Fig. 3a). There were also significant differences in community structure of nematodes and ciliates in response to OM quality (PERMANOVA, Nematodes: Pseudo-F_5,24_ = 1.59, r^2^ = 0.25, p = 0.028; Ciliates: Pseudo-F_5,24_ = 1.88, r^2^ = 0.28, p = 0.001; Table 1, Supplementary Fig. S4). OM treatments significantly affected the relative abundance of the nematodes *Daptonema* (ANOVA, F_5,24_ = 4.68, p = 0.004, Supplementary Table S3) and *Leptolaimus* (ANOVA, F_5,24_ = 6.39, p < 0.001, Supplementary Table S3). The relative abundance of *Daptonema* was higher in 100D compared to 50/50 and 20D/80C, and that of *Leptolaimus* was higher in 100D than in 80D/20C, 50/50 and 20D/80C (Supplementary Table S4). However, these variations in nematode taxonomic assemblages did not translate into significant changes on the proportion of the different nematode feeding types present in our samples (Supplementary Table S3 and Fig. S5). After correcting for multiple testing, none of the ciliate taxonomic groups showed significant variations in relative abundance across treatments (Supplementary Table S3).

OM composition also had a significant effect on bacterial community structure (PERMANOVA, Pseudo-F_5,24_ = 1.36, r^2^ = 0.22, p = 0.003, Table 1), which was mainly driven by the treatment 20D/80C, clustering separately from all other treatments (Fig. 3b). Planctomycetes were more abundant in 20D/80C (2.4 ± 0.3%) than in all other treatments (1.1 ± 0.2–1.6 ± 0.1%) and was the only phylum whose relative abundance differed significantly across treatments (ANOVA, F_5,24_ = 10.04, p < 0.001, Supplementary Table S3).

### Response to OM quality at the ASV and intermediate taxonomic levels

**Figure 4:**
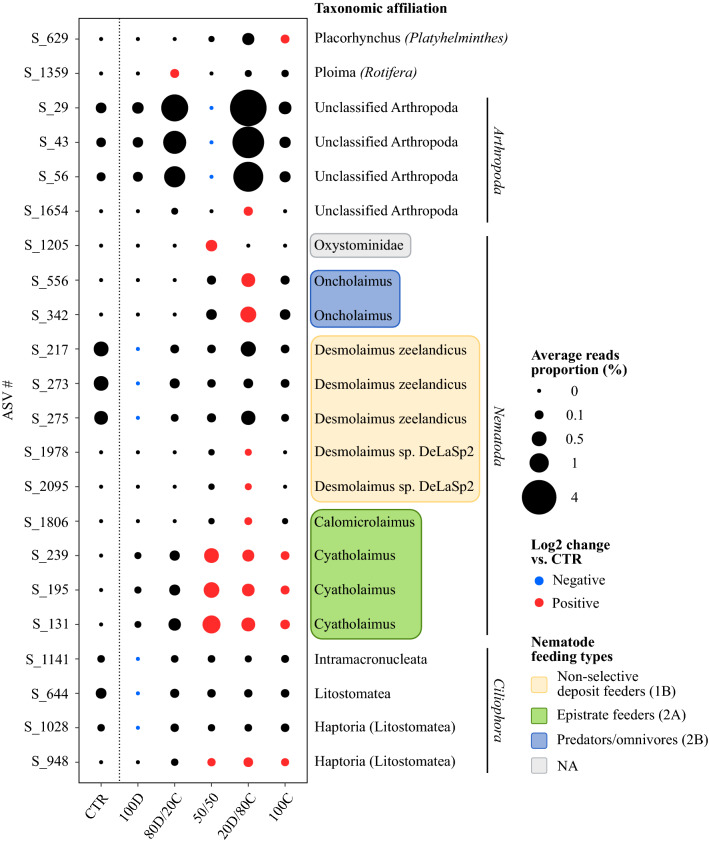
Average relative abundances of 18S rRNA ASVs detected as significantly different in at least one OM treatment compared to control by DESeq2 analysis (increased and decreased abundance displayed in red and blue, respectively. Black bubbles indicate no significant difference compared to control). Unique ASV identifiers are on the left y-axis, and taxonomic affiliation on the right y-axis. The feeding types for nematode ASVs are further indicated by a color-coding. See Fig. 6 for labels on the x-axis.

As revealed by our DESeq2 analyses, the abundance of 22 and 34 ASVs was significantly affected by OM composition in at least one OM treatment compared to CTR in the 18S and 16S rRNA datasets, respectively. Among microeukaryotes (18S rRNA), 13 ASVs increased in abundance in at least one of the cyanobacteria treatments (Fig. 4). In particular, 3 ASVs assigned to *Cyatholaimus* and 1 ASV assigned to *Haptoria* were more abundant in treatments with 50% or more input of cyanobacteria. Other ASVs decreased in abundance in the 100D treatment (6 ASVs, assigned to *Desmolaimus zeelandicus* and ciliates) and 50/50 (3 ASVs assigned to unknown arthropods) compared to CTR (Fig. 4). Among bacteria (16S rRNA), 27 of the 34 ASVs were significantly more abundant in the 20D/80C treatment (Supplementary Fig. S6). These were mostly rare ASVs (< 0.10% total reads), spanning across multiple phyla. The ASVs that contributed most to treatment dissimilarities according to the SIMPER analyses were generally the same ones across different treatments for both the 18S and 16S rRNA datasets, and they accounted for a large portion of total reads (18S: up to 4.9%, 16S: up to 2.0%, Supplementary Fig. S7). Additionally, the abundance of 18 bacterial families was significantly affected by at least one OM treatment. Bacteria affiliated to *Methylomonaceae*, *Unclassified B2M28* (Gammaproteobacteria), *Desulfobulbaceae*, *Desulfobacteraceae* (Deltaproteobacteria), *Bacteroidetes BD2-2* and *Marinifilaceae* (Bacteroidetes) were all enriched in the 100D treatment, and their relative abundance generally increased with increasing proportion of diatoms in OM amendments (Supplementary Fig. S8).

### Denitrification gene expression

**Figure 5:**
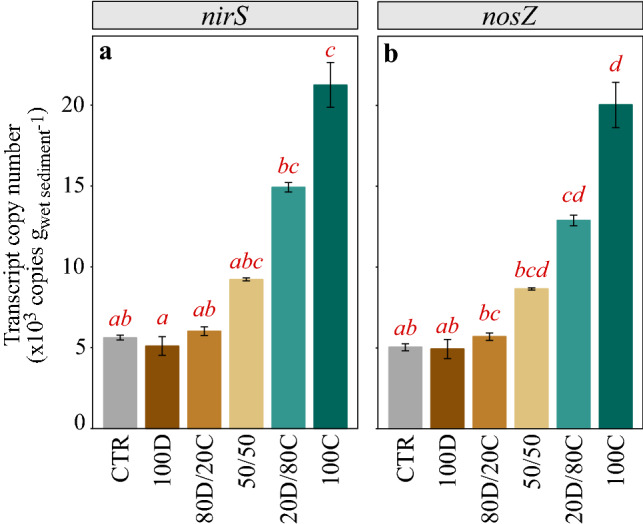
Transcript abundance of the (**a**) *nirS* and (**b**) *nosZ* genes, involved in denitrification (mean ± SE). Transcripts were quantified by real-time quantitative PCR on RNA samples obtained upon experiment termination, and are displayed as gene copy number per g (wet weight) of sediment. Red letter codes designate the significant differences across treatments for each gene based on Kruskal–Wallis and Dunn post-hoc test results. See Fig. 6 for labels on the x-axis.

Expression of the genes *nirS* and *nosZ*, both involved in the denitrification process, displayed a similar response to OM composition, with an overall positive correlation between gene expression and increasing proportions of cyanobacteria in the OM input (KW, p_*nirS*_ < 0.001, p_*nosZ*_ < 0.001, Fig. 5, Table 1). For both genes, the expression level in the 100% diatom treatment was not different from control (Dunn post-hoc, p_nirS,nosZ_ = 1, Table 1), while it was about 4 times higher in the 100% cyanobacteria treatment compared to control (Dunn post-hoc, p_nirS_ = 0.005, p_nosZ_ = 0.002, Table 1).

## Discussion

The quality of settling phytoplankton organic matter (OM) had a significant impact on active microeukaryotic alpha diversity after 4 weeks. Overall, the input of diatom- and cyanobacteria-derived OM appeared to be antagonist in nature, with significant reduction of microeukaryotic alpha diversity in the 100% diatom treatment, contrasting with a slight increase in the treatments with cyanobacteria. Microeukaryotic alpha diversity is generally negatively impacted by high OM loading^20,36,37^, but while OM quality is thought to be equally relevant^3^, its impact on sediment communities is not well-characterized. A number of studies indicate a positive relationship between OM quality (e.g. OM freshness, derived from chl-*a*:phaeo ratio) and benthic fauna diversity indices, although this may vary across different areas^36,38^. Our results bring forward additional evidence on the important role of OM quality in mediating benthic alpha diversity. In particular, the nature of the phytoplankton from which settling OM is derived appears to trigger different responses. For example, the diatom and cyanobacteria OM sources used in our experiment differed in a multitude of parameters including size, shape, elemental composition and stoichiometry. Diatoms had notably low C, N and P content compared to cyanobacteria. Even though the quantity of OM amendment was standardized across our different treatments, the 100C treatment still received ~ 3–4 times more C and N than the 100D treatment. Higher resource availability may explain why cyanobacteria-derived OM appeared to support greater microeukaryotic alpha diversity than diatom amendments^38^. The reason behind the significant decrease in active microeukaryotic diversity in the 100D treatment remains however unclear. In a related experimental set-up, Albert et al*.*^39^ showed a slightly higher sediment O_2_ consumption following spring bloom (i.e. diatoms) compared to summer bloom (i.e. cyanobacteria) amendments. It is possible that a similar oxygen deficiency occurred in our 100D treatment, therefore impacting microeukaryotic diversity negatively^40^, but we lack data to support this hypothesis. Unfortunately, our design did not allow to draw conclusions as to which qualitative features might be driving the alpha diversity patterns observed. Further scrutiny on the impact of individual qualitative features is needed to envision more accurately future benthic response to change in OM supply.

In parallel to changes in alpha diversity, a significant effect of OM quality on active microeukaryotic community structure was observed, although this response was more subtle. The differences in active microeukaryotic community structure across treatments did not appear to be driven by substantial changes at broad taxonomic levels, but rather at the ASV level. Nevertheless our results show that 4 weeks after OM addition, there was a distinction between the microeukaryotic communities in the 100D treatment compared to all the cyanobacteria treatments, regardless of proportion. Spring bloom sedimentation traditionally represents the largest input of OM to the seafloor^5^, and many benthic organisms have adapted to exploit this resource^2,16–18,41^. Yet, not all taxa display a similar response to spring bloom settling, which points to the existence of different strategies in resource utilization^2,17,21,41^. Later in the summer, nitrogen-fixing cyanobacteria can also contribute to OM export towards the sediment, and support benthic secondary production to some extent^6,26,42,43^. Radiotracer experiments have documented that meiofauna can utilize both diatom-^44^ and cyanobacteria-derived OM^26^, albeit with substantial differences in assimilation rates and growth benefits^27^. Although this body of evidence originates from a limited number of taxa, it illustrates that meiofauna taxa differ greatly in their ability to utilize and grow from diatom- and cyanobacteria-derived OM. This can in turn translate to significant changes in community composition in response to OM amendments from these two sources, and partly explain the patterns that were observed in the present study. Nevertheless, the re-organization of the community at broad taxonomic levels (e.g. class, order, even genus) was subtle. Instead, changes in community composition across the OM quality gradient seemed to derive from varying contributions of different ASVs, which could reflect adapted resource utilization among different genotypes^45^. As previously discussed, although with similar C:N ratios, *N. spumigena* had higher C and N content than *S. marinoi*. Microeukaryotic community composition in the 100D treatment could therefore reflect changes in taxa/genotypes able to adapt to this OM elemental composition. Analogously, the input of cyanobacteria OM, even in low proportion, might have supported different taxa/genotypes, better adapted to take advantage of a more labile source of OM^21^. For example, several ASVs that increased in relative abundance with higher proportion of cyanobacteria were assigned to the nematode genus *Cyatholaimus*. Nematodes of the same family, genus *Paracanthoncus*, were previously shown to assimilate C from cyanobacterial blooms at higher rates than other nematodes^26^.

In contrast to a number of previous findings, our results did not show that specific nematode feeding types responded to settling OM quality. Nematodes are typically classified into four feeding types based on their morphology^24^. Due to their small buccal cavity, selective (1A) and non-selective (1B) deposit feeders are thought to feed primarily on bacteria and small detritus particles, while epistrate-feeders (2A) and predators/omnivores (2B) with wider buccal cavities and armatures seem better adapted to feed on microalgae, other meiofauna and protists^11^. In a field study in intertidal habitats, van der Hejden et al*.*^22^ found that the biomass of benthic microalgae and sediment OM were positively related to the relative abundance of epistrate-feeders and non-selective deposit-feeders, respectively. Similarly, we expected a positive influence of all phytoplankton OM treatments on the relative abundance of epistrate-feeders compared to control conditions, but this was not the case in our experiment. The sediment cores, sampled in September, might still have contained post-bloom phytoplankton detritus from the summer and/or spring season^46^, which could have been sufficient to support epistrate-feeders for the duration of the experiment. If these nematodes were not food-limited, the additional input of phytoplankton-derived OM, either diatom or cyanobacteria, would not have promoted an increase in epistrate-feeders relative abundances. The fact that we did not observe a relationship between nematode feeding types and the OM quality gradient could also be explained by feeding plasticity. Indeed, nematodes can modulate their feeding strategies depending on food availability in a way that might not be reflected by their morphological classification^25,44,47,48^.

Analyses of the ciliate community also did not reveal an effect of OM quality on the relative abundance of any particular taxon. Ciliates typically account for a large part of benthic microeukaryotic communities, as described from both morphology- and DNA-based studies^37,41,49^, and their community composition has been shown to respond to OM loading^20,37^. To our knowledge, no studies have specifically addressed the role of OM quality on benthic ciliate community structure. As for meiofauna, ciliate densities display strong seasonal dynamics, which could reflect variations in food resource availability (e.g. bacteria^41^). Indeed, many benthic ciliates feed on bacteria from the sediment or overlying water^50,51^. Stimulation of bacterial production by settling OM could therefore indirectly affect ciliate communities. In a field survey conducted close to our study site, Ankar^41^ demonstrated that nematodes and ciliates had opposite responses to settling OM. While nematode abundance peaked in May, 2 months after the spring bloom (see also Ólafsson and Elmgren^17^), the highest ciliate abundance occurred in September. This suggests that the two groups may not respond to the same environmental drivers, or at least not in the same timeframe, and that ciliates might not directly rely on phytoplankton sedimentation as a food resource in the area. It is worth noting that many ciliate ASVs were not taxonomically identified further than at Order level, which may conceal different species-specific response to the OM quality gradient. With the improvement of DNA reference databases, we will be able to obtain more ecologically meaningful classifications, and better characterize the influence of environmental drivers such as OM quality on ciliate communities.

Active sediment bacterial diversity and community composition after 4 weeks did not show a marked response to phytoplankton-derived OM quality, except in the 20D/80C treatment. In this particular treatment, bacterial alpha diversity increased due to rare ASVs, and a shift in community composition was observed. Our results partly support previous evidence that different algal OM inputs do not necessarily induce major changes in sediment bacterial communities^19,29^. Using a similar experimental set-up to ours but in arctic sediments, Hoffmann et al.^19^ observed little differences in benthic bacterial communities in response to OM amendments from several species of phytoplankton and sea-ice algae. Yet, OM quality in general has been identified as an important structuring factor for sediment bacterial communities^19,30^. Indeed, while some bacteria can utilize a large spectrum of substrates, others are specialized in the exploitation of specific OM sources^52^. Since diatoms and cyanobacteria differ in biochemical composition^27^, we could have expected that the dominance of one or the other OM source may promote the growth of different bacteria taxa^19^. Alternatively, potential effects on bacterivorous microeukaryotes could also have triggered an indirect response of bacterial communities^12,53^. However, we found no clear evidence that certain bacteria taxa (at Phylum or ASV level) were favored by diatoms or cyanobacteria amendments. This could indicate that the active bacterial community at our study site exhibited a large phenotypic plasticity in resource utilization^52^. Similar observations were drawn by Landa et al.^54^ from their experimental study of bacterial communities cultured on diatom- and cyanobacteria-derived dissolved organic matter (DOM), where they did not see changes in communities at a broad taxonomic level in response to either source of DOM. Instead, specific responses may occur at intermediate taxonomic levels^55^, as evidenced by our DESeq2 analyses on bacterial families. Experimental constraints might have also limited our ability to detect clear changes in active sediment bacterial communities in response to OM quality. First, we did not conduct repeated samplings during the 4 weeks of the experiment. Sediment bacteria can respond quickly to fresh OM settling^16^, and we may have missed transient effects that occurred prior to experiment termination. Second, as we sampled the top 3 cm in our sediment cores, we homogenized the upper sediment layer, where changes in active bacteria would have been the clearest, with deeper strata, where bacterial communities may have been more stable after only 4 weeks from OM addition. Finally, we observed a strong positive bottle effect on the Genus *Arcobacter* (Epsilonbacteroata) in all our experimental treatments (17.4 ± 3.7 to 34.8 ± 3.4%, Fig. 1c) compared to T0 (0.4 ± 0.0%, Supplementary Fig. S9). This may have partly masked responses by other bacteria taxa. It remains unclear why active sediment bacterial diversity and community composition in the 20D/80C treatment differ so clearly from all other treatments. Indeed, the availability of mixed food sources—here diatom- and cyanobacteria-derived OM—could promote a greater diversity of heterotroph bacteria, but we would then expect similar effects in the other mixed treatments (50/50 and 80D/20C).

Despite ambiguous responses of bacterial communities to our OM quality gradient, we observed a very clear positive trend of denitrification gene expression (*nirS* and *nosZ*) in response to increasing proportion of cyanobacteria input. Both genes were ~ 4 times more expressed in the 100C than in the 100D treatment, potentially indicating higher denitrification activity in response to cyanobacteria input to the seafloor. It is worth keeping in mind that the link between gene transcripts abundance and biogeochemical rates is not necessarily straightforward, notably for denitrification^56,57^. In addition, due to the short lifespan of messenger RNA (mRNA) transcripts, the measurements taken upon experiment termination only represent a snapshot of the functional response to OM quality. Nevertheless, previous studies did demonstrate increased denitrification rates in response to cyanobacteria amendment^33,39^. As cyanobacteria are typically rich in N, their settlement on the seafloor can lead to mineralization that releases large quantities of dissolved inorganic nitrogen (DIN), and ultimately fuels denitrification^39^.

Continuous advances and decreasing costs of molecular approaches have popularized the use of metabarcoding as a tool for examining marine communities, although important methodological challenges remain^58^. Potential biases introduced during the process should be carefully considered in order to draw meaningful ecological interpretations^59^. First, the limited documentation of DNA barcodes in reference libraries represents an important blind spot in our community analyses. Indeed, 26.7 ± 0.6% and 10.9 ± 0.6% of reads in our 18S and 16S rRNA datasets were not assigned to any known phylum or domain. In addition, the use of a broad reference library such as NCBI *nt* can lead to spurious taxonomic assignments. For example, the nematode *Paracanthonchus *spp., commonly found at the study site in morphologically-based studies^17,27^, was absent from our 18S rRNA dataset, while the genus *Cyatholaimus* genus, one of the most abundant in our dataset, has never been reported in the area. It is possible that, as common members of the Cyatholaimidae family, ASVs from *Paracanthonchus* spp., were incorrectly assigned to *Cyatholaimus*. Second, a common issue in metabarcoding studies has to do with our ability to relate sequence reads to ecologically relevant metrics, such as taxa abundance or biomass^58^. Indeed, rRNA genes may be present in multiple copies in the genome. This is in particular the case for eukaryotes, among which the number of rRNA gene copies can go up to several thousands^60^. Consequently, we obtain a biased representation of the original communities, which may have hampered our capacity to detect changes in taxon abundance or biomass in response to OM quality. Finally, the small sediment volume (2 g) and direct RNA extraction, without prior organisms isolation, were probably sub-optimal for comprehensively examining the microeukaryotic community—notably the metazoan meiofauna—in our sediment cores^61,62^. Despite these methodological drawbacks, rRNA metabarcoding enabled the detection of significant patterns in the response of benthic communities to OM quality, including protists, which are often overlooked in traditional approaches.

In conclusion, this study demonstrates a subtle effect of phytodetritus-derived OM quality on the active benthic microeukaryotic communities. Alpha diversity was negatively impacted by diatom settling, and community structure differed between sediment cores that received only diatoms and those that received some cyanobacteria input. Re-organization of the community was mostly observed at the ASV level, and did not translate to pronounced changes at higher taxonomic levels. Effects on sediment bacterial communities were mostly not very clear, but we did observe a strong stimulation of denitrification gene expression by settling cyanobacteria. Although the latter observation needs to be confirmed by direct denitrification rate measurements, this suggests that functional and structural changes among sediment bacteria may be partly decoupled^63^. Additional work is needed to clarify which OM qualitative feature(s) are driving benthic community responses, but our results indicate that realistic climate-related modifications in the composition of settling OM to aphotic sediments may contribute to changes in benthic community structure and functioning^3^.

## Materials and methods

### Study site and sediment sampling

Sediment cores were collected from Hållsviken, northern Baltic proper (58°50′ N, 17°31′ E) in September 2017. Sampling was carried out at 27-m depth using a box-corer (0.2 m^2^), sub-sampled on board with acrylic cores (30 × 4.6 cm). Thirty-five sediment cores were brought to Askö Marine Research Station, Trosa, Sweden, where they were placed in a thermo-regulated room at in situ temperature (4.5 ± 1 °C). Five cores were used for the characterization of initial conditions in the sediment (T0), following the same procedure as for the experiment termination (see below). The sediment height was adjusted to 10 cm in the remaining cores, and they were topped with brackish water (salinity 6) filtered through a 22 µm sand filter. To ensure constant oxygenation, an air-flow system was connected to each core, after which they were left to equilibrate under faint green light (15:9 h day:night cycle, 0.4 µE m^−2^ s^−1^) until the start of the experiment (i.e. 14 days).

### Organic matter sources

Cultures of the diatom *Skeletonema marinoi* (strain LYS6AAF, provided by the Department of Environmental Science and Analytical Chemistry, Stockholm University) and of the cyanobacterium *Nodularia spumigena* (strain K-1537, provided by the Norwegian Institute for Water Research, Norway) were used to simulate different scenarios of OM sedimentation. Culture conditions are described in Hedberg et al*.*^43^. After 6 days, the phytoplankton material in each culture was rinsed 3 times with sterile artificial seawater and concentrated in a slurry. Simultaneously, salinity of the slurries were gradually brought down to 15 (*S. marinoi*) and 6 (*N. spumigena*) to approximate in situ conditions while preserving the cells integrity (verified visually under a microscope). The slurries were kept in the dark at 10 °C until the start of the experiment (ca. 1 week), and their particulate organic matter (POM) content was estimated as mg_OM_ L^-1^ via loss-on-ignition (*n* = 5, see supplementary method).

### Experimental set-up and termination

**Figure 6:**
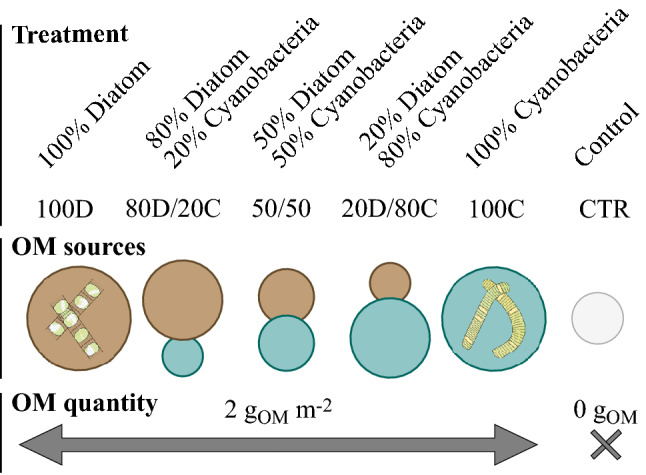
Experimental treatments. Organic matter (OM) quality varied in a gradient from 100% diatoms (*Skeletonema marinoi*) to 100% cyanobacteria (*Nodularia spumigena*), while OM quantity was standardized to 2 g_OM_ m^−2^, as estimated from loss-on ignition. The control treatment (CTR) received no OM input.

Five scenarios of OM input to the sediment were simulated, with varying proportions of diatoms—*S. marinoi*—and cyanobacteria—*N. spumigena :* 100% diatoms (100D), 80% diatoms/20% cyanobacteria (80D/20C), 50% diatoms/50% cyanobacteria (50/50), 20% diatoms/80% cyanobacteria (20D/80C) and 100% cyanobacteria (100C). This was done by adding to each sediment core the relevant ratio of each slurry based on mg_OM_ L^−1^ (Table 2). Total OM added was standardized to 2 g_OM_ m^−2^, an amount within the range of local phytoplankton sedimentation rates^64^. One control treatment with no OM addition was also included. As such, our experiment consisted of six treatments, with five replicate cores each, and OM input ranging from 100% diatoms to 100% cyanobacteria (Fig. 6).

**Table 2:** Organic matter (OM) content and elemental composition of the two plankton slurries and initial sediment (mean ± SE, n = 5).

	Diatoms	Cyanobacteria	Sediment
	*Skeletonema marinoi*	*Nodularia spumigena*	
OM content (mg_OM_ L^−1^)	643.3 ± 45.5	939.9 ± 74.3	–
%OM (%DW)	21.4 ± 0.5	45.1 ± 4.0	–
%C (%DW)	4.2 ± 0.1	26.1 ± 0.5	4.8 ± 0.1
%N (%DW)	0.7 ± 0.01	5.4 ± 0.1	0.6 ± 0.0
%P (%DW)	0.1 ± 0.01	0.2 ± 0.01	0.1 ± 0.0
C:N (mol:mol)	6.8 ± 0.1	5.7 ± 0.03	9.4 ± 0.1
C:P (mol:mol)	80.2 ± 2.0	334.2 ± 17.0	100.9 ± 2.2
N:P (mol:mol)	11.8 ± 0.3	58.9 ± 3.2	10.7 ± 0.3

OM content was estimated from loss-on-ignition. C, N and P content are expressed as % dry weight (DW), ratios are in molar.

Before starting the experiment, the cyanobacteria’s gas vacuoles were collapsed by applying a sudden pressure shock^65^, to allow them to settle to the sediment surface. The air-bubbling was put on hold in the sediment cores, and the diatom and cyanobacteria slurries were homogenized and pipetted evenly at the surface of each sediment core. At this stage, samples from each slurry were preserved at − 20 °C for later elemental composition analyses (see supplementary methods). After 12 h, most of the OM had settled down in the sediment cores, but some cyanobacteria filaments were still floating at the water surface. Buoyant filaments were carefully removed from each core, mixed with 2 g of 500 µm-sieved sediment from the study site, and spread evenly at the sediment surface in the corresponding cores. The same amount of sediment was added to the 100% diatoms and control treatments for homogeneity among treatments. After 24 h, all OM had settled to the sediment, and the sediment cores were covered with parafilm and the air-flow re-started.

The experiment was terminated 27 days after OM addition. The upper 3 cm of sediment were sliced and thoroughly homogenized. Samples for RNA extractions were immediately placed on dry ice, and stored at − 80 °C. Samples for elemental (C, N, P) analyses were preserved on ice and stored at − 20 °C later on (see supplementary methods). All material was autoclaved before starting the experiment, and material and bench thoroughly cleaned with 70% Ethanol between processing of each core to limit cross-contamination.

### RNA extraction and cDNA synthesis

RNA was extracted from the sediment in order to analyze the community composition of biologically active microeukaryotes and bacteria. Total RNA was extracted from 2 g of sediment with RNeasy^®^ PowerSoil^®^ Total RNA Kit (Qiagen), following the manufacturer protocol. RNA concentrations were measured with Nanodrop and standardized to a concentration of 80 ng µL^−1^. RNA extracts were then treated with TURBO DNA-free™ Kit (Invitrogen) to ensure the elimination of DNA traces. Successful DNase treatment was verified through 30-cycle PCR amplification of the 18S rRNA in the RNA extracts, using the same primer pair and PCR conditions as for sequencing preparation, followed by 1% agarose gel electrophoresis. 1 µg of purified RNA was converted to cDNA using the AccuScript High Fidelity 1st Strand cDNA Synthesis Kit (Agilent Technologies), and cDNA products were preserved at − 20 °C until further library preparation for amplicon sequencing.

### Library preparation for 16S and 18S amplicon sequencing

Two amplicon libraries were prepared, targeting the hypervariable regions v4 on the 18S rRNA and v3-v4 on the 16S rRNA, to investigate the microeukaryotic and bacterial community composition respectively. Libraries were prepared independently for the 18S and 16S rRNA barcodes, following a dual-indexed two-step PCR amplification (see detailed protocol in supplementary methods). The libraries were then sequenced on two Illumina MiSeq runs (2 × 300 bp paired-end reads) at the National Genomics Infrastructure (NGI) in Stockholm.

### Bioinformatic pipeline and sequence classification

Raw reads were processed using the DADA2^66^ pipeline (version 1.10.1) implemented in R (version 3.5.1). The sequences were quality-filtered, dereplicated, partitioned into amplicon sequence variants (ASVs), merged, and chimeras were removed to generate final ASV count tables. This was conducted with the following parameters: truncLen = c(240,200) for 18S, c(290,210) for 16S, trimLeft = c(10,10) for 18S, c(8,8) for 16S, maxEE = c(2,2), minFoldParentOverAbundance = 4 and allowoneoff = TRUE. Other parameters were set to default value. Taxonomic assignment of the 18S rRNA ASVs was carried out with the Basic Local Alignment Search Tool (BLAST) against the NCBI *nt* reference database^67^ (January 2019; blastn -evalue 0.001). The full taxonomic classification was then retrieved from the NCBI-taxonomy mapping (nucl_acc2tax_Nov2018) using MEGAN Community Edition software^68^ (version 6.14.2). In order to achieve a better taxonomic resolution for the identification of ciliates, an alignment was also ran against the SILVA database^69^ (release 132) using the function *IdTaxa*^70^ implemented in the DECIPHER package (version 2.10.2). Only sequences identified by the SILVA database were kept for downstream analyses. Finally, ASVs assigned to Bacteria, Archaea, non-target taxa within Eukaryota and ASVs not assigned at the Domain level were discarded (18S_1 and 16S_1, Supplementary Table S1 and S2, see details in supplementary methods). Nematodes were assigned to one of four morphological feeding types based on information in the literature for different genera^17,24,71^. For the 16S rRNA ASVs, taxonomic classification was retrieved from the SILVA database, using the same approach as for the ciliates. ASVs unassigned at Domain or Phylum level, as well as Archaea, Eukaryota and chloroplast sequences were removed from the dataset. In addition, 16S rRNA ASVs present in the phytoplankton slurries but absent from initial (T0) or control (CTR) sediment were also taken out (16S_1, Supplementary Table S2). Following all steps of quality-filtering, 2 ASVs (n = 102 reads, Supplementary Table S2) were detected in the 16S rRNA PCR control. They were also present in other samples, accounting for up to 3.6% of total read abundance. They most likely represent cross-contamination during the library preparation and were therefore not removed from the dataset so as not to lose potentially relevant information.

### Community analyses

Community data analyses and visualization were conducted using the packages phyloseq^72^ (v.1.24.2), vegan^73^ (v.2.5.4) and ggplot2^74^ (v.3.0.0) in R. Rarefaction curves were obtained from the initial 18S and 16S rRNA datasets with the function *rarecurve*. Estimation of alpha diversity were conducted on the filtered datasets (18S_1 and 16S_1, Supplementary Tables S1 and S2) using the Abundance-based Coverage Estimator (ACE) and Shannon’s diversity H′ indices, followed by one-way ANOVAs and TukeyHSD post-hoc tests after ensuring compliance with normality and homoscedasticity assumptions. Alpha diversity was also estimated on rarefied datasets, where read counts were randomly adjusted to the lowest sequencing depth (18S: n = 30,613; 16S: n = 18,452). The patterns detected were the same as for the entire datasets (Supplementary Fig. S10), so the non-rarefied results are presented here. Additionally, alpha diversity of the nematode and ciliate assemblages within the 18S rRNA dataset were estimated. For further analyses, singletons and doubletons (ASVs occurring < 2 times in the dataset) were removed in order to limit the risk of incorporating sequencing artefacts^75^ (18S_2 and 16S_2, Supplementary Tables S1 and S2). Dissimilarity matrices were computed using Sørensen distance to construct Non-metric Multidimensional Scaling (NMDS) ordination plots, based on presence/absence data. After verifying that the dispersion of replicates relative to the group centroid was similar across treatments (PERMDISP2, 9999 permutations, 18S: p = 0.354, Nematodes: p = 0.101, Ciliates: p = 0.508, 16S: p = 0.400), differences in community composition were further investigated with PERMANOVAs using the function *adonis*.

Relative abundances were pooled at genus (18S rRNA, nematodes), order/subclass (18S rRNA, ciliates) and phylum levels (16S rRNA, bacteria). Differences in relative abundances of the most represented groups were tested with one-way ANOVAs or Kruskal–Wallis tests depending on whether the data met the normality and homoscedasticity assumptions. The significance level (α) was adjusted for multiple comparisons by applying a Bonferroni correction (α_adj_ = α/n_tests_) within each dataset (Nematodes, n_tests_ = 11; Nematode feeding types, n_tests_ = 5; Ciliates, n_tests_ = 18; Bacteria, n_tests_ = 12). TukeyHSD post-hoc tests were applied on groups that displayed a significant change in relative abundance.

Changes in individual ASVs (16S and 18S) and families (16S) abundance were estimated using the DESeq2 package^76^ (v.1.22.2) in R. DESeq2 analyses were ran using default settings, identifying ASVs or families whose abundance significantly increased or decreased in the OM treatments compared to CTR (Benjamini-Hochberg-adjusted p values < 0.1). In order to further focus our analyses, ASVs and families that were only absent/present in 1 or 2 replicates in the treatments being compared were excluded (e.g. ASV_1906, present in 0/5 replicate in CTR, and in 1/5 replicate in 50/50, was excluded despite being detected as significantly more abundant in 50/50 (p_adj_ = 0.007)).

Finally, similarity percentages (SIMPER, n_permutations_ = 999) analyses were performed to identify ASVs contributing most to treatment dissimilarities in the 18S and 16S rRNA datasets.

### Quantification of denitrification gene expression

The transcript abundance of two key denitrification genes, *nirS* and *nosZ*, coding for nitrite and nitrous oxide-reductases respectively, were quantified through real-time quantitative PCR (RT-qPCR) following the procedure described in Broman et al.^57^. Briefly, RNA was extracted from 1 g of sediment, and DNA traces removed before conversion to cDNA. RT-qPCR amplifications for *nirS* and *nosZ* were run in triplicates on Bio-Rad C1000 Touch™ Thermal cycler, CFX96™ Real-Time System. Kruskal–Wallis followed by Dunn post-hoc non-parametric tests (Holm correction) were applied to the gene expression data, after averaging the three technical replicate values for each sample.

## Supplementary Information

Supplementary Information.

## Data Availability

The raw sequencing data generated during this experiment has been deposited in the NCBI Sequence Read Archive (SRA) database (http://www.ncbi.nlm.nih.gov/sra) under BioProject PRJNA739856 (BioSample accessions SAMN19804498 and SAMN19804499).
