# Supplementary material for: Phytoplankton settling quality has a subtle but significant effect on sediment microeukaryotic and bacterial communities
Source: Sci Rep. 2021 Dec 15;11:24033. doi: 10.1038/s41598-021-03303-x (PMC8674317; doi:10.1038/s41598-021-03303-x)
Supplement: Supplementary file 1 — Supplementary Information. [file 41598_2021_3303_MOESM1_ESM.pdf]

Supplementary material

Phytoplankton settling quality has a subtle but significant effect on sediment microeukaryotic and bacterial communities

Séréna Albert,*[1] Per Hedberg,[1] Nisha H. Motwani,[2] Sara Sjöling,[2] Monika Winder,[1] Francisco J. A. Nascimento[1,3]

[1]Department of Ecology, Environment and Plant Sciences, Stockholm University, Stockholm, Sweden

[2]Department of Environmental Science, School of Natural Sciences, Technology and Environmental Studies, Södertörn University, Huddinge, Sweden

[3]Baltic Sea Centre, Stockholm University, Stockholm, Sweden

* Correspondence: Séréna Albert, Department of Ecology, Environment and Plant Sciences, Svante Arrhenius 20A, Stockholm University, SE-106 91 Stockholm, Sweden.

serena.albert@su.se

Keywords: OM quality, RNA metabarcoding, microeukaryotes, bacteria, sediment, denitrification

Table of contents

Supplementary methods

Supplementary Figure S1

Supplementary Figure S2

Supplementary Figure S3

Supplementary Figure S4

Supplementary Figure S5

Supplementary Figure S6

Supplementary Figure S7

Supplementary Figure S8

Supplementary Figure S9

Supplementary Figure S10

Supplementary Table S1

Supplementary Table S2

Supplementary Table S3

Supplementary Table S4

Supplementary methods

Plankton slurries loss-on-ignition

For each plankton slurry (diatoms and cyanobacteria), the particulate organic matter (POM) content was estimated via loss-on-ignition, during which the organic portion of the material is combusted at high temperature. Samples of 1 mL were pipetted on pre-combusted GF/F filters, dried at 60°C for 24h, and combusted for 4h at 500°C. The filters were weighed after each step (precision ± 0.001 mg).

Elemental analysis of phytoplankton slurries and sediment

Samples from the phytoplankton slurries were thawed at 4°C overnight and analyzed for C, N and P content. For each slurry, 2 mL-samples were pipetted on pre-weighed, pre-combusted (C/N analyses, n = 5) or regular (P analyses, n = 5) GF/F filters, dried at 60°C for a minimum of 24h, and weighed again. Samples were analyzed for C and N using a Thermo Scientific Flash 2000 Elemental Analyzer, and for P content using an ALPKEM O. I. Analytical Flow Solution IV, following the protocol described in Larsson et al. (2001). Elemental composition is reported as % dry weight.

Sediment samples taken at the start (T0, n = 5) and end of the experiment (n = 30) were dried at 60°C for a minimum of 24h and ground using a mortar and pestle. Sub-samples were then placed in pre-weighed tin capsules for C/N analysis and on GF/F filters for P analysis. The samples were analyzed following the same procedure as for the phytoplankton slurries.

Library preparation for 16S and 18S rRNA

Library preparation consisted of two PCR steps. For the first round of PCR, illumina adapters were attached to the 5' end of the forward and reverse primers for subsequent nested PCR. The primers Bakt_341F (5'-CCTACGGGNGGCWGCAG) and Bakt_805R (5'-GACTACHVGGGTATCTAATCC) were used for 16S rRNA amplification[1] (~460bp amplicon), and the primers TAReuk454FWD1 (5'-CCAGCA(G/C)C(C/T)GCGGTAATTCC), and TAReukREV3 (5'-ACTTTCGTTCTTGAT(C/T)(A/G)A) for 18S rRNA amplification[2] (~380bp amplicon). PCR reactions (25 µL) were done in triplicates using Q5® HS High-Fidelity 2X Master Mix (New England BioLabs), 0.2µM of each primer and 1µL of cDNA template (diluted 1:10); negative PCR controls were ran simultaneously using molecular-grade water. The PCR-1 conditions comprised an initial denaturation step of 30 s at 98°C, followed by 15 cycles of 10 s at 98°C, 30 s at 50°C, 30 s at 72°C for the 18S barcode (12 cycles for 16S), and a final elongation step of 10 min at 72°C. Residual dNTPs and primers were then cleaned out from the round-1 PCR products using a mix of 0.1µL Exonuclease I (New England BioLabs) and 0.2µL Thermosensitive Alkaline Phosphtase (TSAP, Promega), incubated 15 min at 37°C and inactivated 15 min at 74°C. The products of this enzymatic reaction were used as templates for the second round of PCR amplification. The PCR-2 conditions were the same for the 18S and

16S library preparation: 3 min at 95°C, followed by 15 cycles of 30 s at 95°C, 30 s at 55°C, 30 s at 72°C, and a final step of 5 min at 72°C. The primers used for PCR-2 comprised short index sequences, and each sample was amplified using a unique combinations of forward and reverse indexed-primers. PCR triplicates were then pooled and purified using Agencourt AMPure XP (Beckman Coulter) magnetic beads. Qubit® 2.0 Fluorometer (with Qubit® dsDNA BR Assay Kit, Invitrogen) was used to quantify the amplicon products, before standardizing and pooling the samples in equimolar amounts. The final 16S rRNA library pool was further loaded on a 1.5% agarose gel, the DNA fragment corresponding to the amplicons was excised and purified using the GeneJET Gel Extraction Kit (Thermo Scientific).

18S data filtering

The following Eukaryota taxa were removed from the 18S rRNA dataset, since they were not targeted in our study: Cryptophyta, Viridiplantae, Haptophyceae, Rhodophyta, Stramenopiles, Dinophyceae, Fungi, Deuterostomia (common carp *C. carpio* or unclassified), Gymnoplea (pelagic copepods *E. affinis*, *A. tonsa* and *A. bifilosa*) and Keratella (pelagic rotifer *K. quadrata*).

References

1. Herlemann, D. P. R. *et al.* Transitions in bacterial communities along the 2000 km salinity gradient of the Baltic Sea. *ISME J.* **5**, 1571–1579 (2011).

2. Stoeck, T. *et al.* Multiple marker parallel tag environmental DNA sequencing reveals a highly complex eukaryotic community in marine anoxic water. *Mol. Ecol.* **19**, 21–31 (2010).

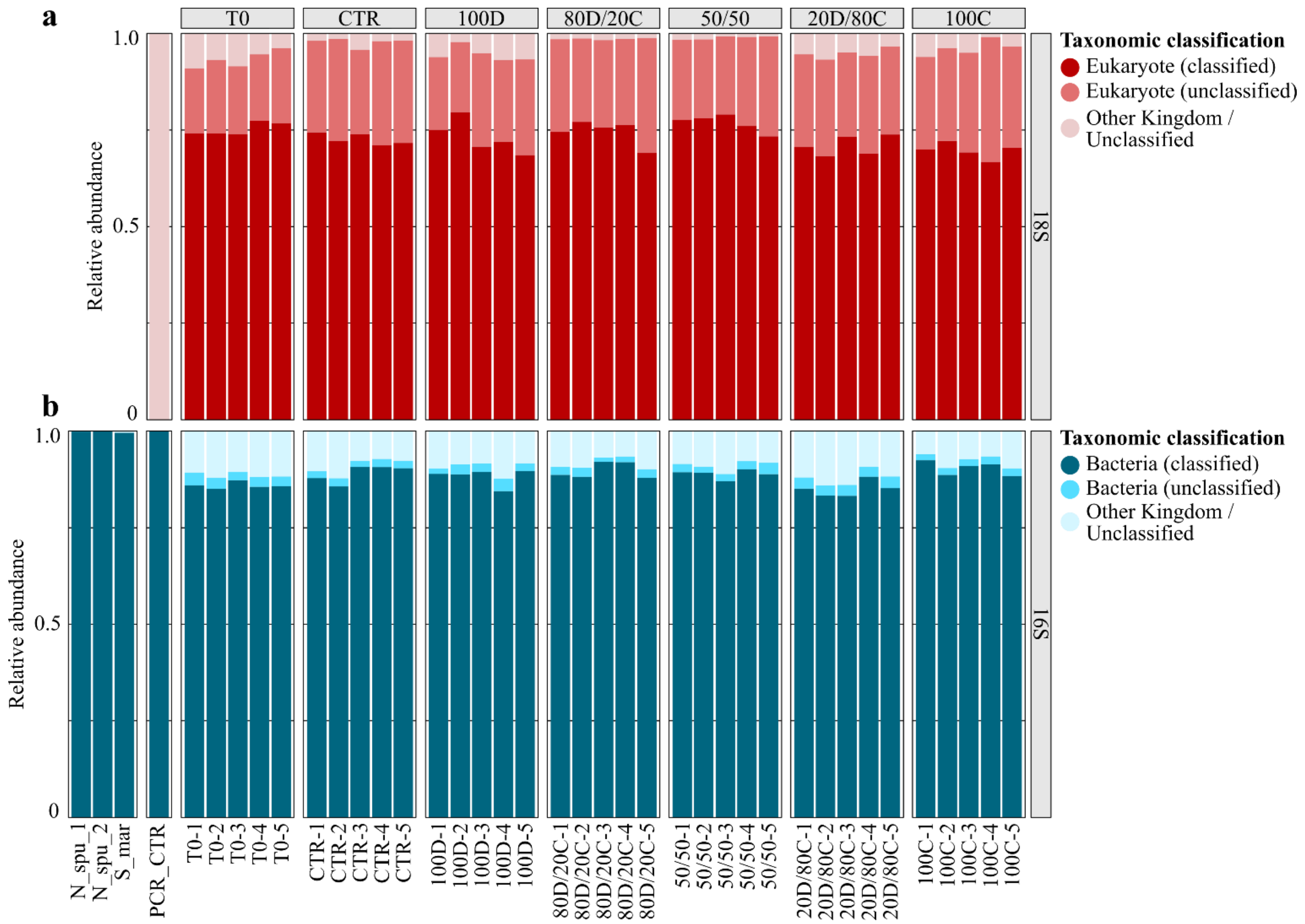

Figure S1. Proportion of reads classified at Domain level within the (a) microeukaryotes (18S rRNA) and (b) bacteria (16S rRNA) datasets. Reads attributed to either target domains but not identified at phylum level are displayed as Eukaryote (unclassified) and Bacteria (unclassified). See Fig.6 for labels on the x-axis. N_spu = cyanobacteria slurry, S_mar = diatom slurry, PCR_CTR = negative PCR control.

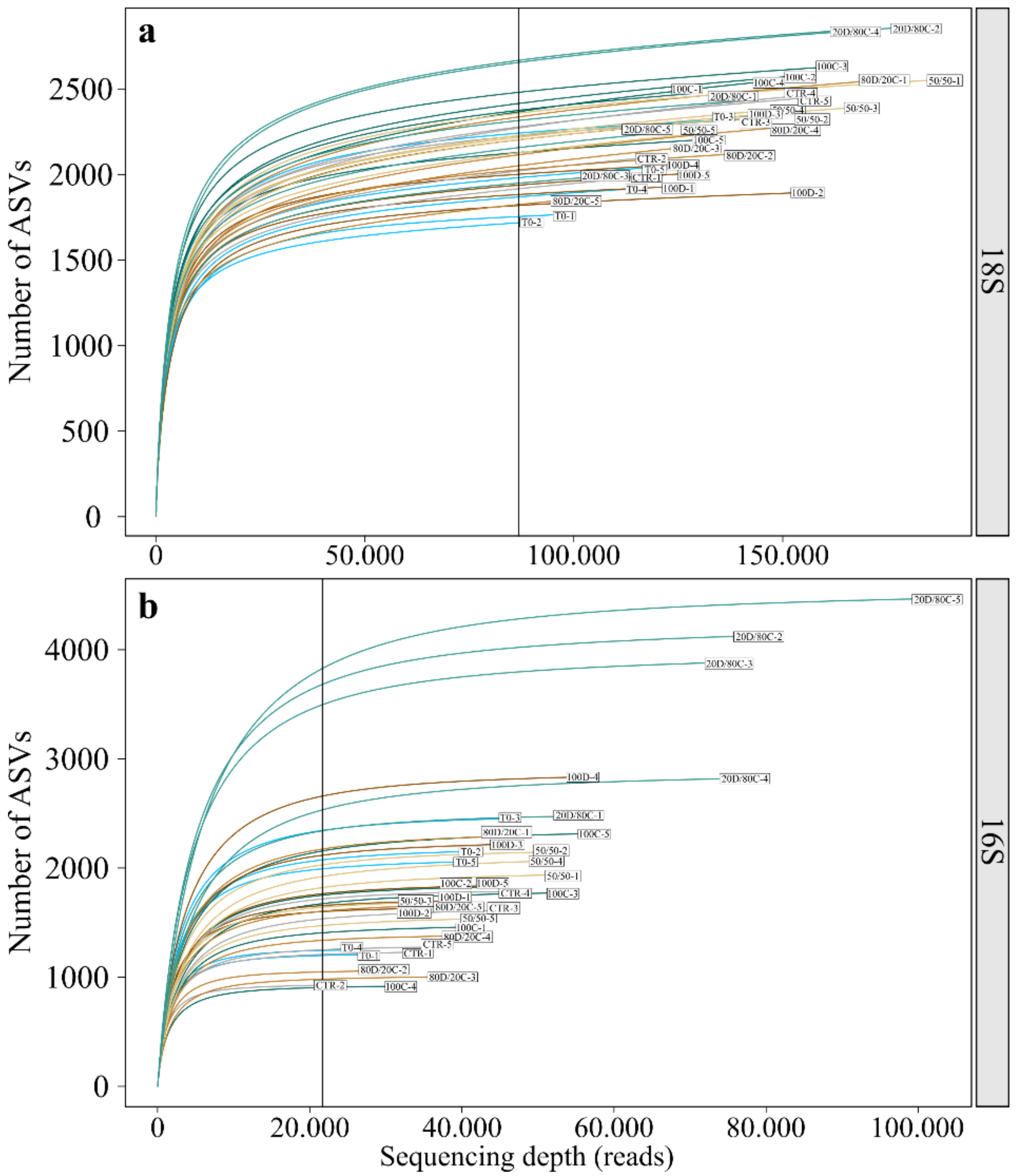

Figure S2. Rarefaction curves displaying the number of ASVs obtained as a function of sequencing depth for the (a) microeukaryotes (18S rRNA) and (b) bacteria (16S rRNA) datasets. The vertical lines indicate the sample with lowest sequencing depth, used to rarefy each dataset.

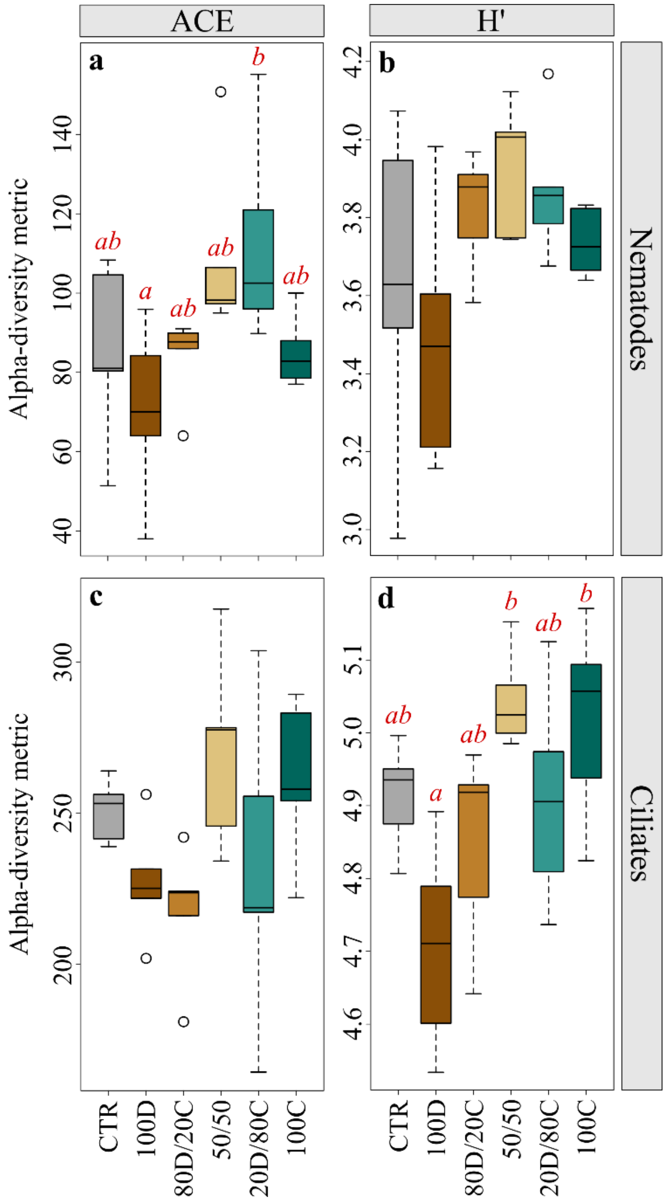

Figure S3. Alpha diversity indices (Abundance-based coverage estimator (ACE) and Shannon's diversity index H') for (a, b) nematodes and (c, d) ciliates upon experiment termination (18S rRNA dataset). Red letter codes designate the significant differences across treatments based on one-way ANOVA and TukeyHSD post-hoc test results. See Fig.6 for labels on the x-axis.

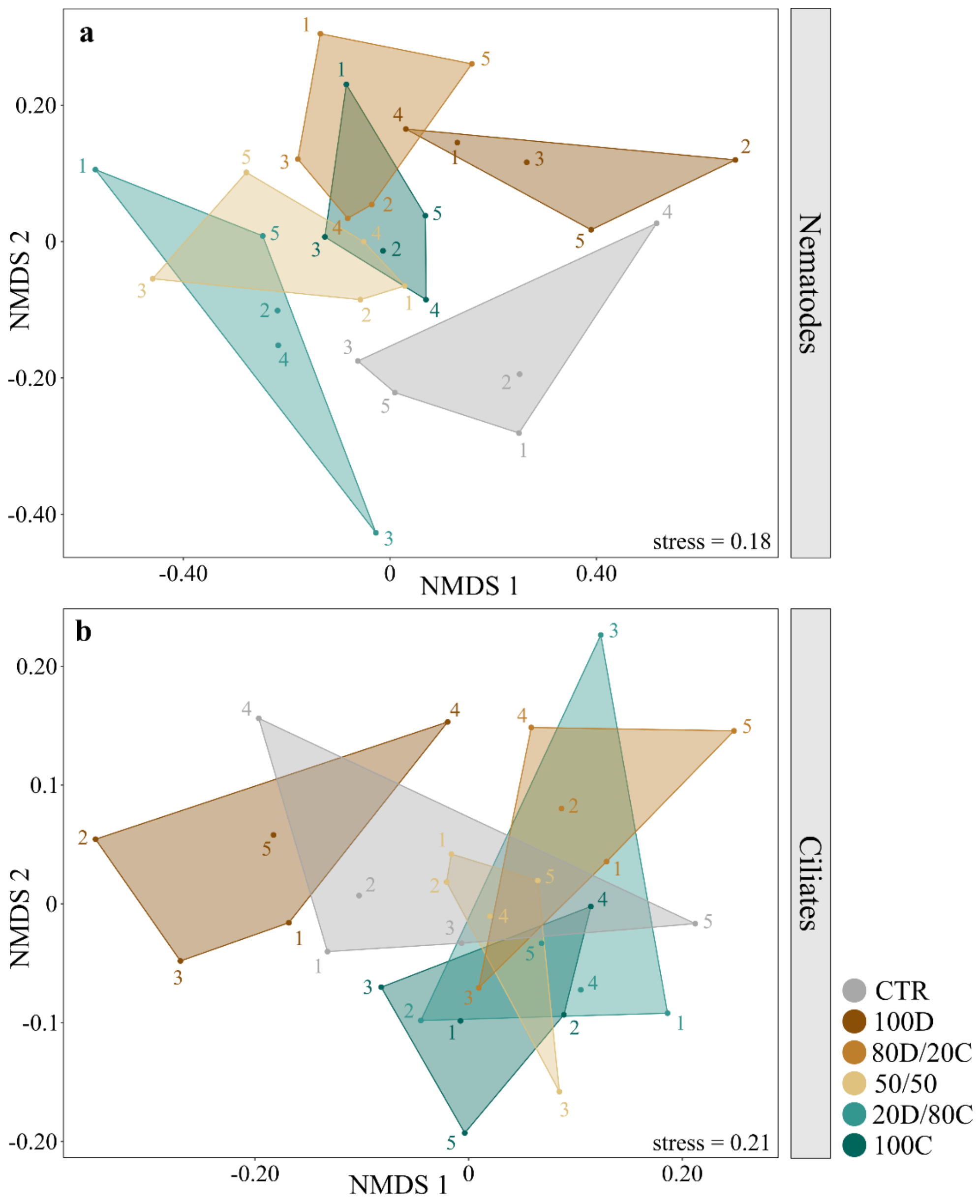

Figure S4. NMDS ordination plots based on Sørensen distance matrices of (a) nematodes and (b) ciliate communities, calculated from 18S rRNA ASVs presence/absence, respectively. The stress value for each ordination is displayed in the bottom right corner. See Fig.6 for treatment codes in the legend.

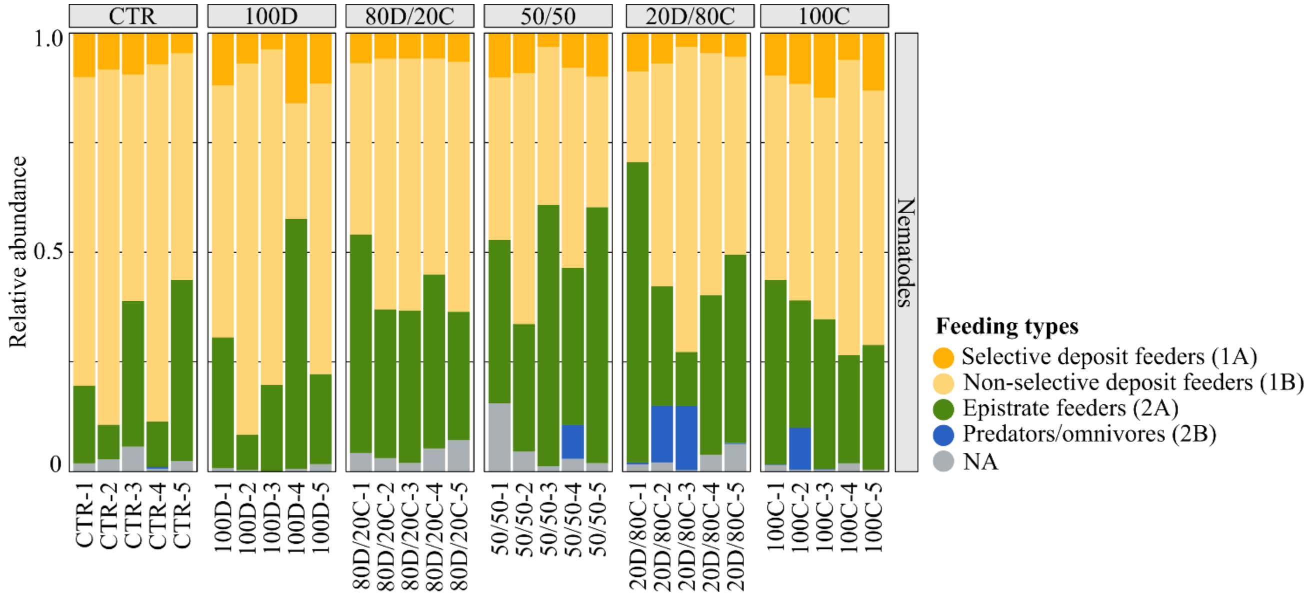

Figure S5. Relative abundance of nematode feeding types retrieved from taxonomic classification of 18S rRNA. Nematodes for which taxonomic classification was not precise enough to accurately attribute a feeding type were classified as NA. See Fig.6 for labels on the x-axis.

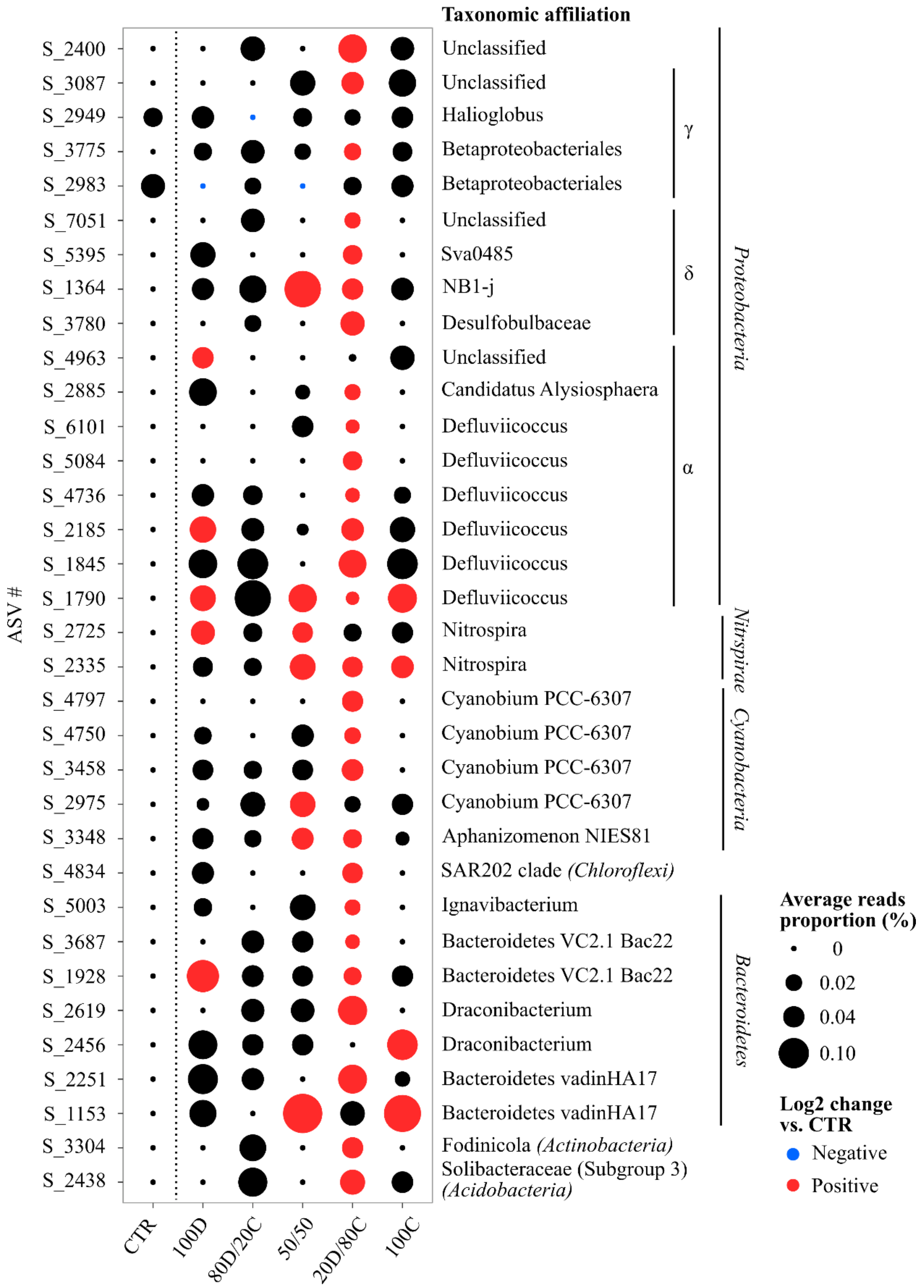

Figure S6. Average relative abundances of 16S rRNA ASVs detected as significantly different in at least one OM treatment compared to control by DESeq2 analysis (increased and decreased abundance displayed in red and blue, respectively. Black bubbles indicate no significant difference compared to control). Unique ASV identifiers are on the left y-axis, and taxonomic affiliation on the right y-axis. See Fig.6 for labels on the x-axis.

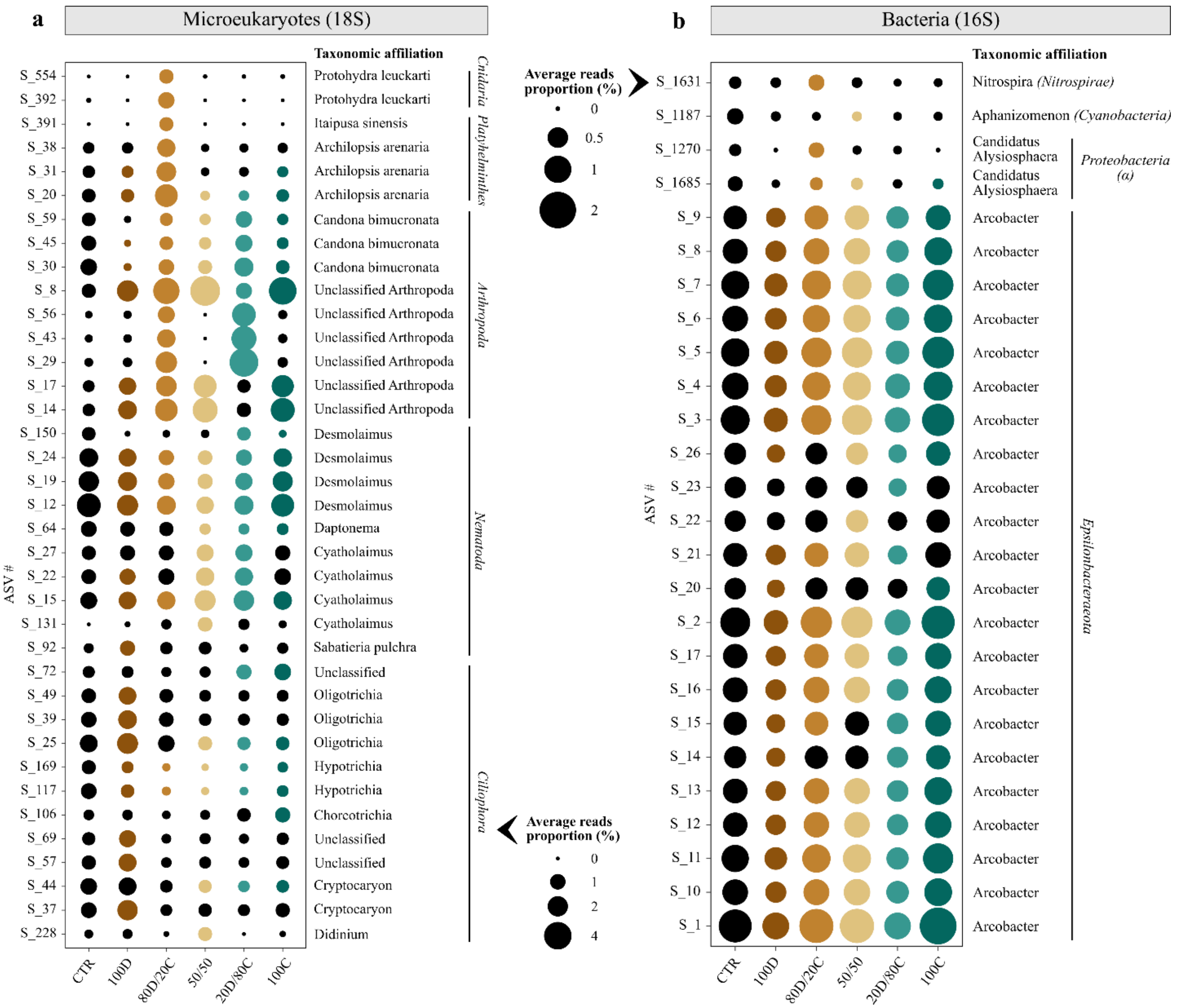

Figure S7. Average relative abundances of the top 20 (a) 18S rRNA and (b) 16S rRNA ASVs detected as contributing most to dissimilarities in each treatment by SIMPER analysis. ASVs are color-coded if they contributed to dissimilarities between a particular treatment compared to control, otherwise, they are displayed in black. Unique ASV identifiers are on the left y-axis, and taxonomic affiliation on the right y-axis. See Fig.6 for labels on the x-axis.

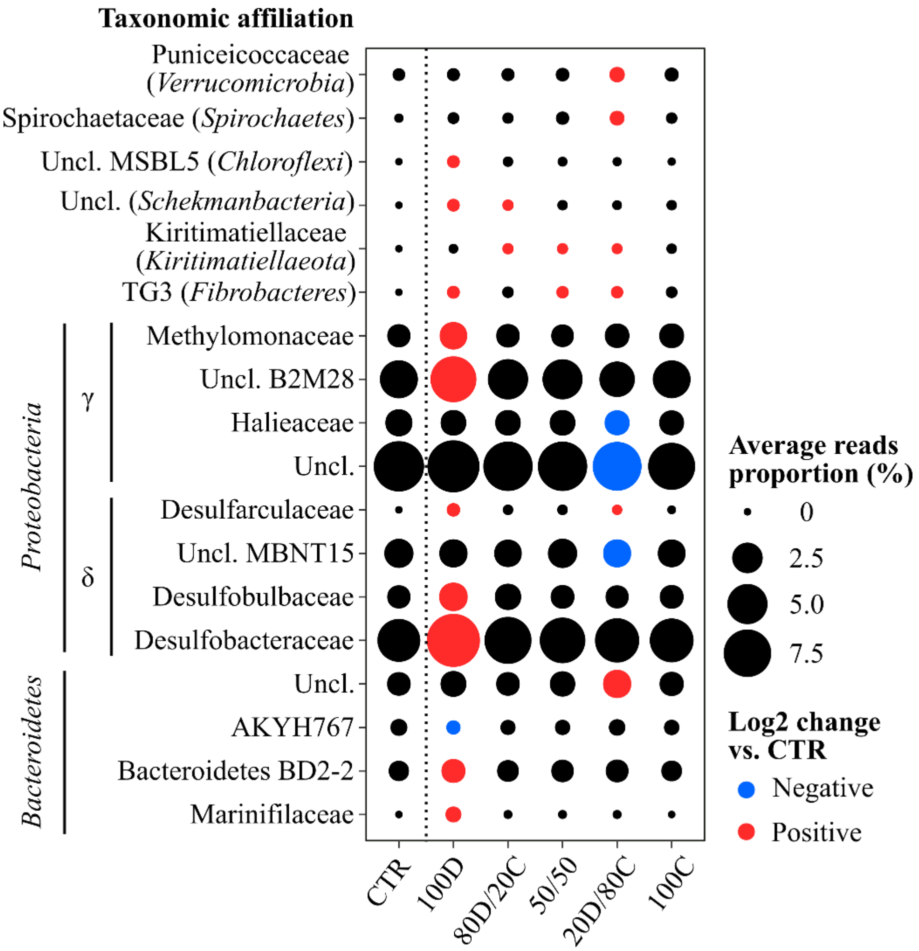

Figure S8. Average relative abundances of 16S rRNA families detected as significantly different in at least one OM treatment compared to control by DESeq2 analysis (increased and decreased abundance displayed in red and blue, respectively. Black bubbles indicate no significant difference compared to control). See Fig.6 for labels on the x-axis.

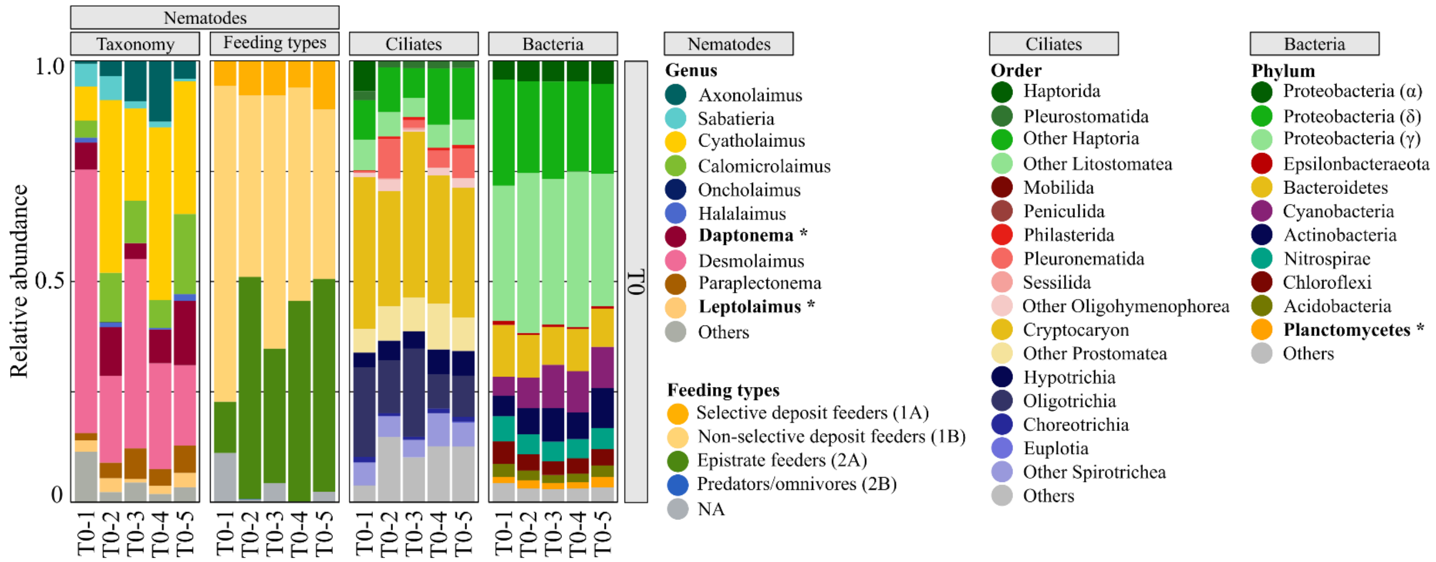

Figure S9. Taxonomic composition of nematodes (18S rRNA, genus level and feeding types), ciliates (18S rRNA, order level) and bacterial (16S rRNA, phylum level) communities in the initial sediment (T0), displayed as ASVs relative abundances. See Fig.6 for labels on the x-axis.

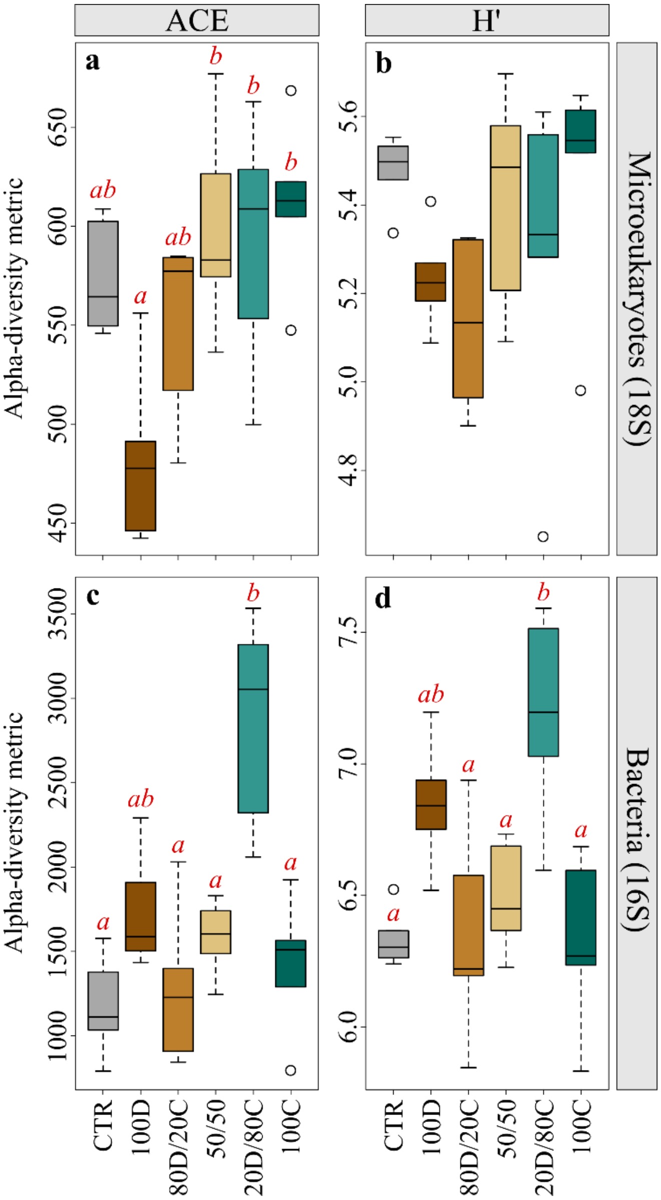

Figure S10. Alpha diversity indices (Abundance-based coverage estimator (ACE) and Shannon's diversity index H') on rarefied datasets for (a, b) microeukaryotes and (c, d) bacteria upon experiment termination. Red letter codes designate the significant differences across treatments based on one-way ANOVA and TukeyHSD post-hoc test results. See Fig.6 for labels on the x-axis.

Table S1. Number of reads and ASVs at different stages of the 18S rRNA dataset processing. The initial dataset was used to produce the rarefaction plots. The dataset 18S_1 was further filtered to include only microeukaryotic target taxa, and was used to estimate alpha diversity metrics. The dataset 18S_2 was further trimmed from single- and doubletons, and was used for community composition analyses. See Fig.6 for treatment labels.

	Initial dataset (18S)		18S_1		18S_2	
Sample	Reads	ASVs	Reads	ASVs	Reads	ASVs
PCR_CTR	-	-	-	-	-	-
T0-1	95,669	1,765	30,541	341	30,524	329
T0-2	86,802	1,718	29,743	425	29,719	406
T0-3	139,695	2,326	44,228	541	44,196	517
T0-4	115,558	1,919	54,034	471	54,005	449
T0-5	116,365	2,038	50,894	556	50,857	523
CTR-1	116,947	1,984	40,553	548	40,526	529
CTR-2	117,365	2,099	40,966	570	40,928	538
CTR-3	144,685	2,328	54,156	610	54,119	582
CTR-4	152,148	2,457	45,221	578	45,165	532
CTR-5	153,704	2,445	47,776	628	47,726	586
100D-1	121,850	1,927	40,951	498	40,939	490
100D-2	155,131	1,895	41,677	451	41,662	439
100D-3	145,468	2,339	48,859	566	48,819	534
100D-4	118,468	2,049	40,208	458	40,193	443
100D-5	125,371	2,004	43,996	484	43,968	463
80D/20C-1	170,314	2,547	62,056	616	62,009	578
80D/20C-2	136,906	2,119	50,021	536	49,984	510
80D/20C-3	123,635	2,152	51,825	601	51,781	565
80D/20C-4	148,420	2,274	63,503	622	63,429	564
80D/20C-5	95,425	1,846	30,613	482	30,589	461
50/50-1	187,569	2,555	54,020	613	53,982	580
50/50-2	152,747	2,355	51,991	557	51,964	532
50/50-3	165,905	2,390	72,697	707	72,648	664
50/50-4	151,659	2,386	54,298	652	54,248	606
50/50-5	127,390	2,233	44,182	582	44,147	552
20D/80C-1	141,690	2,435	60,563	646	60,509	602
20D/80C-2	177,608	2,859	58,629	679	58,596	651
20D/80C-3	113,283	2,002	47,945	512	47,898	474
20D/80C-4	168,311	2,850	53,203	661	53,160	625
20D/80C-5	122,564	2,249	44,907	570	44,874	541
100C-1	128,166	2,474	43,668	611	43,625	580
100C-2	152,993	2,577	59,676	644	59,635	609
100C-3	159,294	2,627	54,701	687	54,652	645

100C-4	147,808	2,550	47,062	625	47,000	574
100C-5	129,331	2,202	42,162	566	42,132	540
**Total**	**4,806,204**	**19,361**	**1,701,525**	**4,102**	**1,700,208**	**3,079**

Table S2. Number of reads and ASVs at different stages of the 16S rRNA dataset processing. The initial dataset was used to produce the rarefaction plots. The dataset 16S_1 was further filtered to include only bacteria target taxa, and was used to estimate alpha diversity metrics. The dataset 16S_2 was further trimmed from single- and doubletons, and was used for community composition analyses. Ske_T0 and Nod_T0 refer to *Skeletonema marinoi* and *Nodularia spumigena s*lurry samples, respectively. See Fig.6 for treatment labels.

	Initial dataset (16S)		16S_1		16S_2	
Sample	Reads	ASVs	Reads	ASVs	Reads	ASVs
PCR_CTR	102	2	-	-	-	-
Ske_T0	232,964	1,688	-	-	-	-
Nod_T0_1	344,727	905	-	-	-	-
Nod_T0_2	349,852	853	-	-	-	-
T0-1	26,390	1,206	22,587	1,068	22,567	1,054
T0-2	39,614	2,149	33,501	1,908	33,461	1,879
T0-3	45,685	2,452	39,695	2,242	39,658	2,214
T0-4	24,708	1,249	21,049	1,105	21,033	1,094
T0-5	38,882	2,054	33,202	1,848	33,152	1,810
CTR-1	33,285	1,228	29,065	1,057	29,053	1,046
CTR-2	21,654	926	18,452	793	18,435	779
CTR-3	43,899	1,610	39,658	1,418	39,614	1,385
CTR-4	45,485	1,785	41,036	1,624	41,025	1,616
CTR-5	35,188	1,275	31,635	1,138	31,606	1,114
100D-1	36,920	1,695	32,699	1,530	32,671	1,510
100D-2	33,914	1,629	29,973	1,481	29,950	1,463
100D-3	43,743	2,214	38,999	1,984	38,982	1,970
100D-4	54,432	2,832	45,839	2,395	45,796	2,363
100D-5	42,284	1,836	37,771	1,628	37,747	1,609
80D/20C-1	46,865	2,295	41,317	2,067	41,275	2,036
80D/20C-2	26,878	1,055	23,606	924	23,595	914
80D/20C-3	35,455	1,002	32,447	878	32,419	855
80D/20C-4	39,867	1,378	36,544	1,251	36,509	1,225
80D/20C-5	37,043	1,659	32,373	1,455	32,348	1,436
50/50-1	50,967	1,935	45,396	1,687	45,375	1,670
50/50-2	50,547	2,146	44,834	1,911	44,820	1,899
50/50-3	35,627	1,687	30,786	1,503	30,765	1,484
50/50-4	50,074	2,062	44,769	1,805	44,738	1,778
50/50-5	42,724	1,536	37,741	1,309	37,714	1,288
20D/80C-1	52,861	2,473	44,741	2,143	44,703	2,114
20D/80C-2	75,745	4,122	62,239	3,523	62,145	3,452
20D/80C-3	71,954	3,878	57,553	3,223	57,453	3,148
20D/80C-4	73,885	2,817	64,770	2,424	64,702	2,371

20D/80C-5	102,872	4,470	87,143	3,769	87,046	3,695
100C-1	41,310	1,460	38,042	1,331	38,014	1,308
100C-2	41,930	1,825	36,671	1,585	36,630	1,554
100C-3	51,210	1,770	46,443	1,570	46,421	1,553
100C-4	31,016	917	28,132	802	28,118	791
100C-5	55,249	2,313	48,435	2,026	48,391	1,990
**Total**	**2,507,807**	**22,023**	**1,379,143**	**15,017**	**1,377,931**	**14,097**

Table S3. Summary of statistical tests used to evaluate differences in relative abundance of target groups within the nematodes, ciliates and bacteria datasets. Either one-way ANOVAs or Kruskal-Wallis (KW) tests were applied, and significance level ($\alpha = 0.05$) was adjusted for multiple comparisons within each dataset ($\alpha_{adj} = \alpha / n_{tests}$). Significant p-values ($< \alpha_{adj}$) are indicated in bold.

	α_{adj}	Target group	Test	Statistic	p
**Nematodes**	**0.0045**	Axonolaimus	KW	$H_{(5)} = 6.18$	0.289
		Sabatieria	KW	$H_{(5)} = 9.92$	0.077
		Cyatholaimus	KW	$H_{(5)} = 9.42$	0.093
		Calomicrolaimus	ANOVA	$F_{(5,24)} = 1.21$	0.334
		Oncholaimus	KW	$H_{(5)} = 12.01$	0.035
		Halalaimus	KW	$H_{(5)} = 6.98$	0.222
		**Daptonema**	ANOVA	$F_{(5,24)} = 4.68$	**0.004**
		Desmolaimus	ANOVA	$F_{(5,24)} = 2.94$	0.033
		Paraplectonema	KW	$H_{(5)} = 6.68$	0.246
		**Leptolaimus**	ANOVA	$F_{(5,24)} = 6.39$	**< 0.001**
		Others	KW	$H_{(5)} = 16.76$	0.005
**Nematode feeding types**	**0.01**	Selective deposit-feeders (1A)	ANOVA	$F_{(5,24)} = 2.50$	0.059
		Non-selective deposit-feeders (1B)	ANOVA	$F_{(5,24)} = 2.07$	0.105
		Epistrate feeders (2A)	ANOVA	$F_{(5,24)} = 1.48$	0.234
		Predators / Omnivores (2B)	KW	$H_{(5)} = 12.01$	0.035
		NA	KW	$H_{(5)} = 14.41$	0.013
**Ciliates**	**0.0028**	Haptorida	KW	$H_{(5)} = 10.50$	0.062
		Pleurostomatida	KW	$H_{(5)} = 8.32$	0.139
		Other Haptoria	ANOVA	$F_{(5,24)} = 2.28$	0.078
		Other Litostomatea	KW	$H_{(5)} = 7.97$	0.158
		Mobilida	KW	$H_{(5)} = 5.00$	0.416
		Peniculida	KW	$H_{(5)} = 2.05$	0.842
		Philasterida	ANOVA	$F_{(5,24)} = 0.61$	0.694
		Pleuronematida	KW	$H_{(5)} = 6.77$	0.239
		Sessilida	KW	$H_{(5)} = 2.55$	0.769
		Other Oligohymenophorea	KW	$H_{(5)} = 8.12$	0.150
		Cryptocaryon	KW	$H_{(5)} = 9.66$	0.086
		Other Prostomatea	KW	$H_{(5)} = 0.80$	0.977
		Hypotrichia	KW	$H_{(5)} = 5.41$	0.368
		Oligotrichia	KW	$H_{(5)} = 15.70$	0.008

		Choreotrichia	KW	$H_{(5)} = 8.60$	0.126
		Euplotia	KW	$H_{(5)} = 6.13$	0.294
		Other Spirotrichea	ANOVA	$F_{(5,24)} = 2.72$	0.044
		Others	ANOVA	$F_{(5,24)} = 3.02$	0.030
**Bacteria**	**0.0042**	Alphaproteobacteria	ANOVA	$F_{(5,24)} = 2.40$	0.067
		Deltaproteobacteria	ANOVA	$F_{(5,24)} = 2.24$	0.083
		Gammaproteobacteria	ANOVA	$F_{(5,24)} = 3.26$	0.022
		Epsilonbacteraeota	ANOVA	$F_{(5,24)} = 2.21$	0.086
		Bacteroidetes	ANOVA	$F_{(5,24)} = 3.49$	0.016
		Cyanobacteria	ANOVA	$F_{(5,24)} = 1.66$	0.183
		Actinobacteria	ANOVA	$F_{(5,24)} = 1.04$	0.415
		Nitrospirae	ANOVA	$F_{(5,24)} = 0.66$	0.655
		Chloroflexi	ANOVA	$F_{(5,24)} = 2.78$	0.040
		Acidobacteria	ANOVA	$F_{(5,24)} = 3.00$	0.030
		**Planctomycetes**	ANOVA	$F_{(5,24)} = 10.04$	**< 0.001**
		Others	ANOVA	$F_{(5,24)} = 4.17$	0.007

Table S4. Results of the TukeyHSD post-hoc tests for the taxonomic groups that showed significant differences in relative abundance across treatments. The treatment groupings are displayed as letter codes. See Fig.6 for treatment labels.

	Taxo group	p	CTR	100D	80D_20C	50_50	20D_80C	100C
Nematodes	Daptonema	**0.004**	*ab*	*a*	*ab*	*b*	*b*	*ab*
	Leptolaimus	**< 0.001**	*ab*	*a*	*b*	*b*	*b*	*ab*
Bacteria	Planctomycetes	**< 0.001**	*a*	*a*	*a*	*a*	*b*	*a*
